# Supplementary material for: Psychometric properties of the polish version of the Dysfunctional Thoughts about Caregiving Questionnaire (DTCQ)
Source: PLoS One. 2025 May 9;20(5):e0320850. doi: 10.1371/journal.pone.0320850 (PMC12063841; doi:10.1371/journal.pone.0320850)
Supplement: S1 File — (PDF) [file pone.0320850.s001.pdf]

Supplementary Materials - DTCQ

Psychometric Properties of the Polish Version of the Dysfunctional Thoughts About Caregiving Questionnaire

Katarzyna Sanna[1]¶*, Maciej Kościelniak[2] ¶, Jarosław M Michałowski[3]&, Andrés Losada-Baltar[4]&, Konrad Piotrowski[1]¶

[1]Center for Research on Personality Development, Institute of Psychology, SWPS University, Poznań, Poland

[2] Institute of Psychology, SWPS University, Poznań,Poland

[3]Poznan Laboratory of Affective Neuroscience, Institute of Psychology, SWPS University, Poznań,Poland

[4] Department of Psychology, Universidad Rey Juan Carlos, Madrid, Spain

Contents:

1. **Descriptive statistics of the studied variables**
2. **Descriptive statistics of the studied items**
3. **One-Factor Model**
   - **Model Fit Indices**
   - **Model Diagram**
   - **Table of Unstandardized and Standardized Factor Loadings with R-square Values**
4. **Two-Factor Model**
   - **Model Fit Indices**
   - **Model Diagram**
   - **Table of Unstandardized and Standardized Factor Loadings with R-square Values**

This document contains supplementary materials for the article published in PLOS ONE

Table 1. Descriptive statistics of the studied variables

	N	Min	Max	M	SD	Sk	*β2*
DTCQ Total	618	0	64	29.07	11.81	.16	-.52
DTCQ Responsibility	618	0	40	17.67	7.71	.15	-.54
DTCQ Perfectionism	618	0	24	11.40	4.73	.12	-.53
UMICS Commitment	618	0	25	14.58	4.36	-.06	-.39
UMICS In-depth Exploration	618	5	25	18.01	3.37	-.79	1.18
UMICS Reconsideration of Commitments	618	3	15	6.22	2.53	.67	-.19
MSPSS Friends	618	0	28	20.38	5.5	-1.09	1.09
MSPSS Family	618	0	28	20.13	5.97	-1.01	.66
MSPSS Significant person	618	0	28	21.28	5.48	-1.11	1.52
MSPSS Total	618	0	84	61.79	14.40	-1.15	2.18
Depression total	618	0	25.	12.54	4.76	.69	-.07

Note. DTCQ = Dysfunctional Thoughts About Caregiving Questionnaire; SCL-27 = Symptom Checklist-27+ Questionnaire; MSPSS = Multidimensional Scale of Perceived Social Support; U-MICS = Utrecht-Management of Identity Commitments Scale; Min = Minimum; Max = Maximum; M = Mean; SD = Standard Deviation;Sk = Skewness; *β2* = Kurtosis.

Table 2. Descriptive statistics of the DTCQ items

Item	M	SD	Sk	*β2*	Min	Max
DTCQ1	1.710	1.206	0.265	-1.019	0.000	4.000
DTCQ2	1.696	1.147	0.207	-0.968	0.000	4.000
DTCQ3	1.683	1.143	0.265	-1.032	0.000	4.000
DTCQ4	2.015	1.165	0.002	-1.073	0.000	4.000
DTCQ5	2.199	1.325	-0.114	-1.192	0.000	4.000
DTCQ6	1.638	1.005	0.285	-0.654	0.000	4.000
DTCQ7	1.754	1.112	0.206	-0.883	0.000	4.000
DTCQ8	2.283	1.098	-0.328	-0.820	0.000	4.000
DTCQ9	2.023	1.129	-0.186	-1.067	0.000	4.000
DTCQ10	1.845	1.167	0.188	-1.095	0.000	4.000
DTCQ11	2.079	1.144	-0.194	-1.007	0.000	4.000
DTCQ12	1.356	1.071	0.668	-0.400	0.000	4.000
DTCQ13	1.877	1.189	0.059	-1.191	0.000	4.000
DTCQ14	1.544	1.147	0.414	-0.889	0.000	4.000
DTCQ15	1.413	1.061	0.558	-0.513	0.000	4.000
DTCQ16	1.956	1.103	0.014	-1.029	0.000	4.000

Note. Min = Minimum; Max = Maximum; M = Mean; SD = Standard Deviation;Sk = Skewness; *β2* = Kurtosis.

One-factor model

Model Fit Indices

The one-factor CFA model for the DTCQ scale (N = 618) demonstrated acceptable fit according to conventional criteria:

- Chi-square test: $\chi^2(104) = 255.310$, $p < .001$
- Root Mean Square Error of Approximation (RMSEA) = 0.049, 90% CI [0.041, 0.056]
- Comparative Fit Index (CFI) = 0.948
- Tucker-Lewis Index (TLI) = 0.940
- Standardized Root Mean Square Residual (SRMR) = 0.036

The RMSEA value below 0.05 suggests close fit, with the upper bound of the confidence interval not exceeding 0.06. Both CFI and TLI values are close to 0.95, indicating good fit. The SRMR value below 0.08 also supports adequate model fit.

Diagram 1

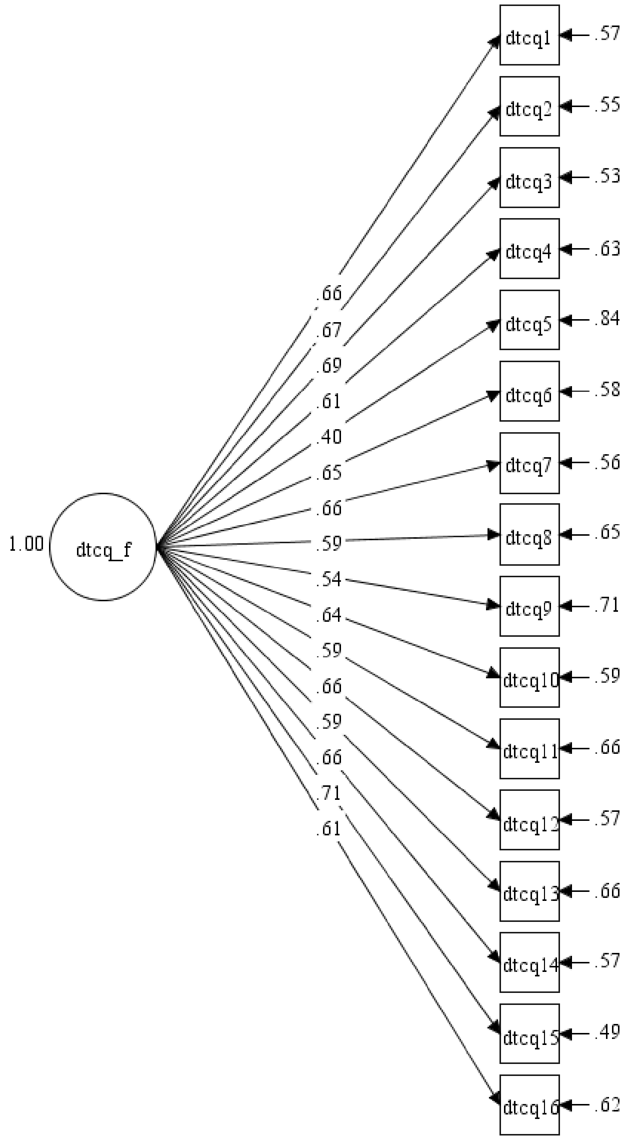

Factor Loadings and R-square Values

Table S1. Unstandardized (B) and Standardized (β) Factor Loadings and R-square Values for the One-Factor DTCQ Model

Item	B (SE)	β (SE)	p-value	R^2 (SE)
DTCQ1	1.000 (-)	0.655 (0.028)	< .001	0.429 (0.036)
DTCQ2	0.978 (0.062)	0.674 (0.028)	< .001	0.454 (0.038)
DTCQ3	0.992 (0.057)	0.685 (0.027)	< .001	0.469 (0.037)
DTCQ4	0.901 (0.061)	0.611 (0.029)	< .001	0.373 (0.036)
DTCQ5	0.671 (0.071)	0.400 (0.037)	< .001	0.160 (0.030)
DTCQ6	0.823 (0.056)	0.647 (0.028)	< .001	0.418 (0.036)
DTCQ7	0.931 (0.062)	0.661 (0.028)	< .001	0.437 (0.037)
DTCQ8	0.819 (0.060)	0.589 (0.030)	< .001	0.347 (0.035)
DTCQ9	0.774 (0.059)	0.542 (0.031)	< .001	0.293 (0.034)
DTCQ10	0.943 (0.067)	0.638 (0.030)	< .001	0.408 (0.038)
DTCQ11	0.850 (0.065)	0.587 (0.031)	< .001	0.344 (0.036)
DTCQ12	0.890 (0.061)	0.656 (0.028)	< .001	0.431 (0.037)
DTCQ13	0.884 (0.066)	0.587 (0.031)	< .001	0.344 (0.037)
DTCQ14	0.953 (0.059)	0.656 (0.026)	< .001	0.430 (0.034)
DTCQ15	0.960 (0.057)	0.715 (0.024)	< .001	0.511 (0.034)

DTCQ16	0.858 (0.058)	0.615 (0.031)	< .001	0.378 (0.039)

Note: B = unstandardized coefficient; β = standardized coefficient; SE = Standard Error; R^2 = amount of variance explained in each item by the laAtent factor. All estimates were obtained using MLR estimation. The unstandardized loading for DTCQ1 was fixed to 1.0 for scale identification.

Two-factor model

Model Fit Indices

The two-factor CFA model for the DTCQ scale (N = 618) demonstrated acceptable fit according to conventional criteria:

- Chi-square test: $\chi^2(103) = 248.069$, $p < .001$
- Root Mean Square Error of Approximation (RMSEA) = 0.048, 90% CI [0.040, 0.055]
- Comparative Fit Index (CFI) = 0.950
- Tucker-Lewis Index (TLI) = 0.942
- Standardized Root Mean Square Residual (SRMR) = 0.036

The RMSEA value below 0.05 suggests close fit, with the upper bound of the confidence interval not exceeding 0.06. Both CFI and TLI values are above 0.94, indicating good fit. The SRMR value below 0.08 also supports adequate model fit.

Diagram 2:

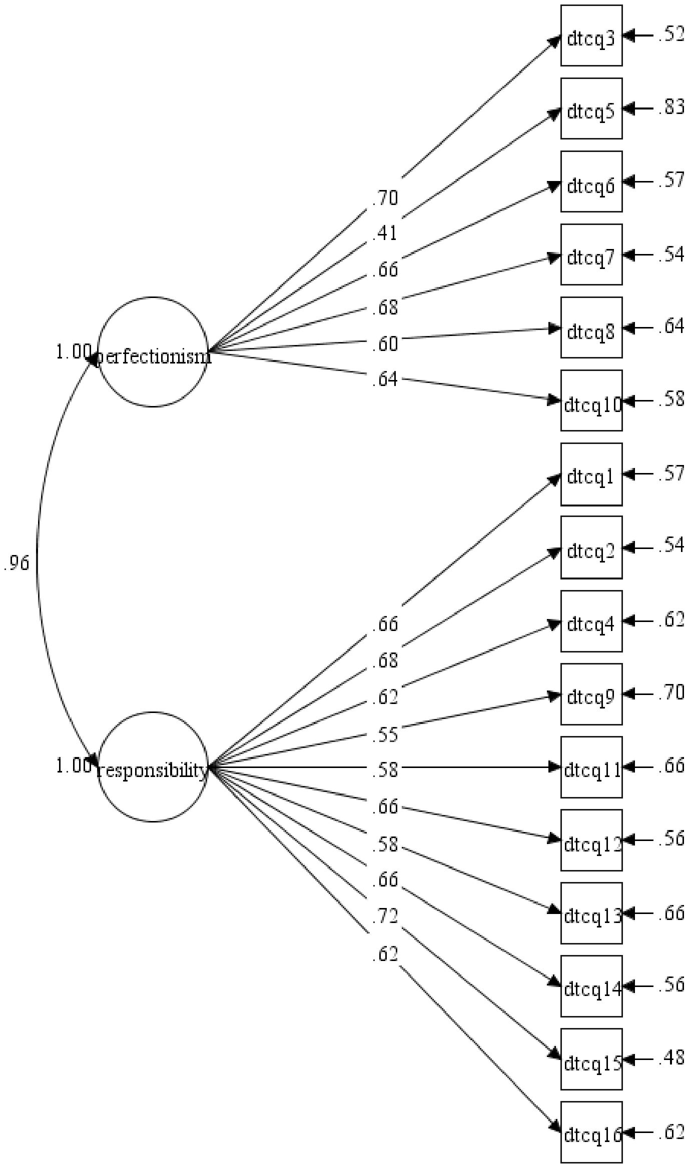

Factor Loadings and R-square Values

Table S2. Unstandardized (B) and Standardized (β) Factor Loadings and R-square Values for the Two-Factor DTCQ Model

Perfectionism Factor

**Item**	**B (SE)**	**p-value**	**β (SE)**	**p-value**	**R^2 (SE)**	**p-value**
**DTCQ3**	1.000 (-)	-	0.696 (0.028)	< .001	0.485 (0.039)	< .001
**DTCQ5**	0.679 (0.070)	< .001	0.408 (0.038)	< .001	0.166 (0.031)	< .001
**DTCQ6**	0.832 (0.054)	< .001	0.659 (0.027)	< .001	0.434 (0.036)	< .001
**DTCQ7**	0.944 (0.058)	< .001	0.676 (0.028)	< .001	0.457 (0.038)	< .001
**DTCQ8**	0.830 (0.055)	< .001	0.602 (0.030)	< .001	0.362 (0.036)	< .001
**DTCQ10**	0.944 (0.066)	< .001	0.644 (0.030)	< .001	0.415 (0.039)	< .001

Responsibility Factor

**Item**	**B (SE)**	**p-value**	**β (SE)**	**p-value**	**R^2 (SE)**	**p-value**
**DTCQ1**	1.000 (-)	-	0.659 (0.028)	< .001	0.434 (0.037)	< .001
**DTCQ2**	0.975 (0.061)	< .001	0.676 (0.029)	< .001	0.457 (0.039)	< .001
**DTCQ4**	0.903 (0.061)	< .001	0.616 (0.029)	< .001	0.380 (0.036)	< .001
**DTCQ9**	0.780 (0.059)	< .001	0.549 (0.031)	< .001	0.301 (0.034)	< .001
**DTCQ11**	0.841 (0.065)	< .001	0.584 (0.032)	< .001	0.341 (0.037)	< .001
**DTCQ12**	0.889 (0.061)	< .001	0.659 (0.028)	< .001	0.435 (0.038)	< .001
**DTCQ13**	0.876 (0.066)	< .001	0.585 (0.032)	< .001	0.342 (0.037)	< .001

**DTCQ14**	0.954 (0.059)	< .001	0.661 (0.026)	< .001	0.437 (0.034)	< .001
**DTCQ15**	0.961 (0.058)	< .001	0.720 (0.024)	< .001	0.518 (0.034)	< .001
**DTCQ16**	0.859 (0.057)	< .001	0.619 (0.031)	< .001	0.383 (0.039)	< .001

Note: B = unstandardized coefficient; β = standardized coefficient; SE = Standard Error; R^2 = amount of variance explained in each item by the latent factor. All estimates were obtained using MLR estimation. The unstandardized loading for DTCQ3 (Perfectionism) and DTCQ1 (Responsibility) were fixed to 1.0 for scale identification.

Factor Correlation

The correlation between Perfectionism and Responsibility factors was 0.961 (SE = 0.016, $p < .001$).
