# Supplementary material for: Psychometric properties of the polish version of the Dysfunctional Thoughts about Caregiving Questionnaire (DTCQ)
Source: PLoS One. 2025 May 9;20(5):e0320850. doi: 10.1371/journal.pone.0320850 (PMC12063841; doi:10.1371/journal.pone.0320850)
Supplement: S2 File — (PDF) [file pone.0320850.s002.pdf]

Kwestionariusz Dysfunkcjonalnych Myśli Dotyczących Opieki

Adaptacja:
Katarzyna Sanna
Konrad Piotrowski

Centrum Badań nad Rozwojem Osobowości, Uniwersytet SWPS
Poznań
kontakt: ksanna@swps.edu.pl & konrad.piotrowski@swps.edu.pl

Instrukcje: Proszę wskazać, na ile zgadza się Pan/ Pani się z każdym stwierdzeniem w skali:

0 Całkowicie się nie zgadzam
1 Nie zgadzam się
2 Ani się zgadzam, ani się nie zgadzam
3 Zgadzam się
4 Całkowicie się zgadzam

		Całkowicie się nie zgadzam	Nie zgadzam się	Ani się zgadzam ani się nie zgadzam	Zgadzam się	Całkowicie się zgadzam
1	Tylko najbliższy członek rodziny osoby chorej naprawdę wie jak się nim/nią zająć	0	1	2	3	4
2	Samolubstwem jest poświęcenie przez opiekuna czasu dla siebie, w czasie gdy bliska osoba jest słaba/chora i potrzebuje opieki	0	1	2	3	4
3	Bycie dobrym opiekunem oznacza nie popełnianie błędów podczas pełnienia opieki nad słabym/chorym bliskim	0	1	2	3	4
4	Dobry opiekun to taki, który pomaga swojemu bliskiemu we wszystkich zadaniach, również tych, które bliski może wykonać sam, jeśli miałoby to bliskiemu ułatwić życie	0	1	2	3	4
5	To byłoby niewybaczalne, gdyby opiekun pomyślał, że „byłoby lepiej dla wszystkich, gdyby mój bliski zmarł”	0	1	2	3	4
6	Jeśli opiekun odczuwa zakłopotanie i odrzucenie w stosunku do swojego bliskiego, to dzieje się tak, ponieważ opiekun w jakiś sposób nie wypełnia swoich obowiązków	0	1	2	3	4

	opiekuńczych					
7	Dobrzy opiekunowie powinni przez cały dzień pozostawać szczęśliwi i w dobrym nastroju, aby odpowiednio radzić sobie z codziennymi zadaniami opiekuńczymi	0	1	2	3	4
8	Dobry opiekun nigdy nie powinien złościć się lub tracić kontroli w stosunku do osoby, którą się opiekuje	0	1	2	3	4
9	Logiczne jest, że opiekunowie rezygnują z własnych potrzeb, odkładając na bok własne zadowolenie z życia na rzecz potrzeb swoich bliskich	0	1	2	3	4
10	Opiekun powinien szukać pomocy u innych tylko wtedy, gdy nie wie, jak rozwiązać problem	0	1	2	3	4
11	Bez względu na to, jak źle się czuje opiekun, nie powinien dawać upustu swoim emocjom przy innych, ponieważ byłby to brak szacunku dla osoby, którą się opiekuje	0	1	2	3	4
12	Opiekunowie powinni unikać rozmawiania o swoich problemach z innymi, ponieważ inni mają własne życie i nie trzeba im zawracać głowy kolejnymi problemami	0	1	2	3	4
13	Opiekun powinien szukać pomocy u innych lub znajdować inne sposoby radzenia sobie tylko wtedy, gdy sytuacja opieki jest w najgorszym punkcie lub gdy nie może już sobie z nią poradzić	0	1	2	3	4
14	Proszenie o pomoc osób spoza rodziny jest ostatnią rzeczą, jaką powinien zrobić opiekun, ponieważ opieką nad słabym/chorym krewnym powinna zająć się rodzin	0	1	2	3	4
15	Kiedy osoba opiekuje się słabą/chorą bliską osobą,	0	1	2	3	4

	powinna odłożyć na bok swoje zainteresowania i całkowicie poświęcić się opiece					
16	Jako opiekun czuję, że powinienem robić wszystko, o co prosi mnie mój słaba/chora bliska osoba, nawet jeśli uważam, że jest to nadmiernie wymagające	0	1	2	3	4
