# Supplementary material for: Psychometric properties of the polish version of the Dysfunctional Thoughts about Caregiving Questionnaire (DTCQ)
Source: PLoS One. 2025 May 9;20(5):e0320850. doi: 10.1371/journal.pone.0320850 (PMC12063841; doi:10.1371/journal.pone.0320850)
Supplement: S3 File — (PDF) [file pone.0320850.s003.pdf]

Dysfunctional Thoughts About Caregiving Questionnaire (Losada, 2005; Losada, Montorio, Izal y Márquez-González, 2006)

Description: The Dysfunctional Thoughts about Caregiving Questionnaire (DTCQ) (Losada, 2005; Losada, Montorio, Izal, & Márquez-González, 2006) has been developed as a tool for identifying specific beliefs in caregivers of dependent elderly people that may act as barriers or obstacles for coping adaptively with caregiving. The DTCQ consists of 16 items rated on a 5-point Likert scale ranging from 0=totally disagree to 4 totally agree (range: 0 to 64; higher scores indicate more barriers or obstacles). It has good psychometric properties such as Cronbach's alpha of .89, test-retest reliability of .60 and a correlation of .58 with the Dysfunctional Attitudes Scale (Weissman & Beck, 1978).

Instructions: Please indicate your agreement with each statement on a scale of

0 Totally disagree
1 Disagree
2 Neither agree nor disagree
3 Agree
4 Totally agree

1	Only the closest person to the frail/sick older adult knows how to truly take care of him or her.
2	It is selfish for a caregiver to dedicate time to himself/herself when a relative is frail/sick and needs care.
3	To become a good caregiver would mean not making mistakes when taking care of a frail/sick relative.
4	A good caregiver is one that helps his/her relative with all tasks, including those that the relative can do for himself/herself, if it makes life easier for the relative.
5	It would be unforgivable for a caregiver to think that "it would be better for everyone if my relative died".
6	If a caregiver has feelings of embarrassment and rejection toward his/her relative, it's because the caregiver is failing in some form with his/her caregiving duties.
7	Good caregivers should remain happy and in good spirits all day long to deal adequately with the daily tasks of caregiving.
8	A good caregiver should never get mad or loose control with the person that is being cared for.
9	It is logical for caregivers to give up their own needs, setting aside their own life satisfaction, in favor of their relative's needs.
10	A caregiver should only seek help from others when he/she does not know how to solve a problem.

11	No matter how badly a caregiver feels, he/she should not vent with others because it would be disrespectful to the person being cared for.
12	Caregivers should avoid talking about their problems with others because others have their own lives and don't need to be bothered with more problems.
13	A caregiver should only seek help from others or find other alternatives when the caregiving situation is at its worst or when he/she can no longer handle it.
14	Asking for help from persons who are not part of the family is the last thing that a caregiver should do because caring for a frail/sick relative should be handled by the family.
15	When a person takes care of a frail/sick relative, he/she should set aside his/her interests, and dedicate himself/herself completely to the care of the frail/sick relative.
16	As a caregiver, I feel that I should do everything that my frail/sick relative asks me to do, even though I might believe it is excessively demanding.

Published in:

Losada, A. (2005). *Influencia de los pensamientos disfuncionales sobre el cuidado en el malestar psicológico de cuidadores de personas mayores con demencia. Resultados de un estudio transversal y de intervención*. [Influence of dysfunctional thoughts about caregiving on dementia caregivers´ psychological distress. Results of a correlational and an intervention study]. Doctoral Dissertation: Universidad Autónoma de Madrid.

Losada, A., Montorio, I., Izal, M. y Márquez-González, M. (2006). *Estudio e intervención sobre el malestar psicológico de los cuidadores de personas con demencia. El papel de los pensamientos disfuncionales.* Madrid: IMSERSO .

Acknowledgementes: The authors are grateful to Bob G. Knight and Gia Shurgot-Robinson (University of Southern California, Andrus Gerontology Center) for their valuable comments and help in the DTCQ back-translation.

For more information, please contact andres.losada@urjc.es
