# Supplementary figures and images for: Psychometric properties of the polish version of the Dysfunctional Thoughts about Caregiving Questionnaire (DTCQ)
Source: PLoS One. 2025 May 9;20(5):e0320850. doi: 10.1371/journal.pone.0320850 (PMC12063841; doi:10.1371/journal.pone.0320850)

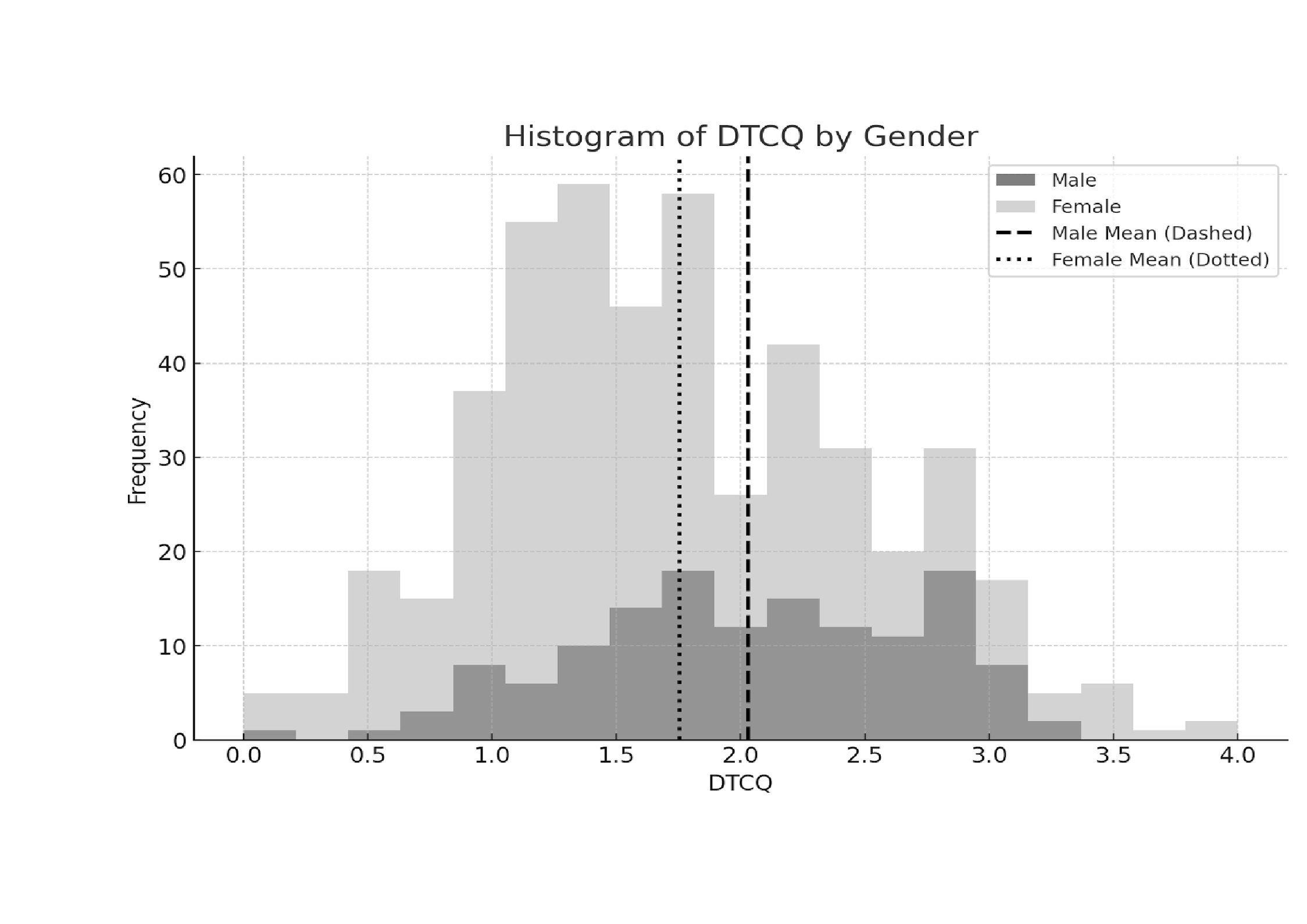

Supplement: S1 Fig — (TIF) [file pone.0320850.s004.tif]

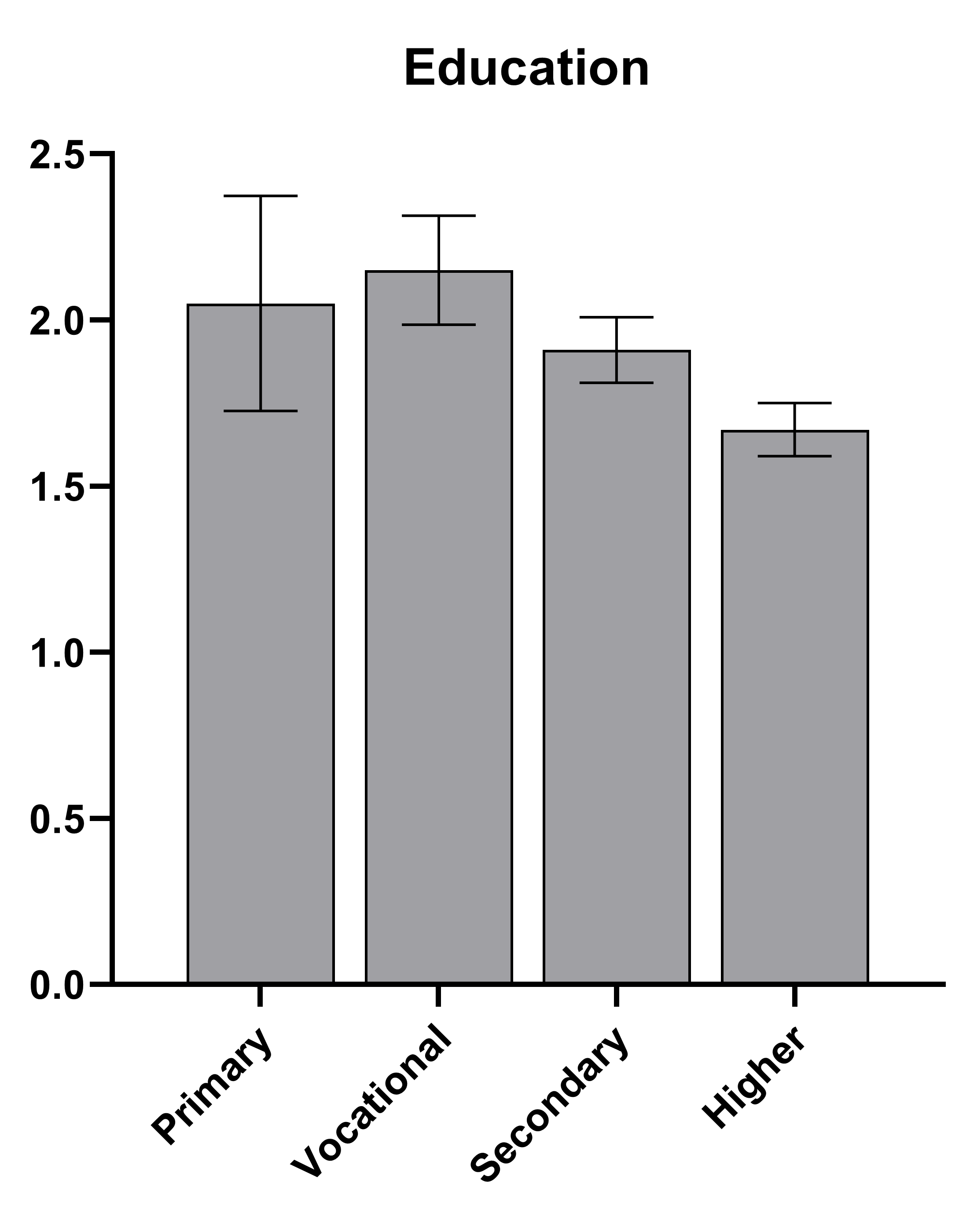

Supplement: S2 Fig — (TIF) [file pone.0320850.s005.tif]

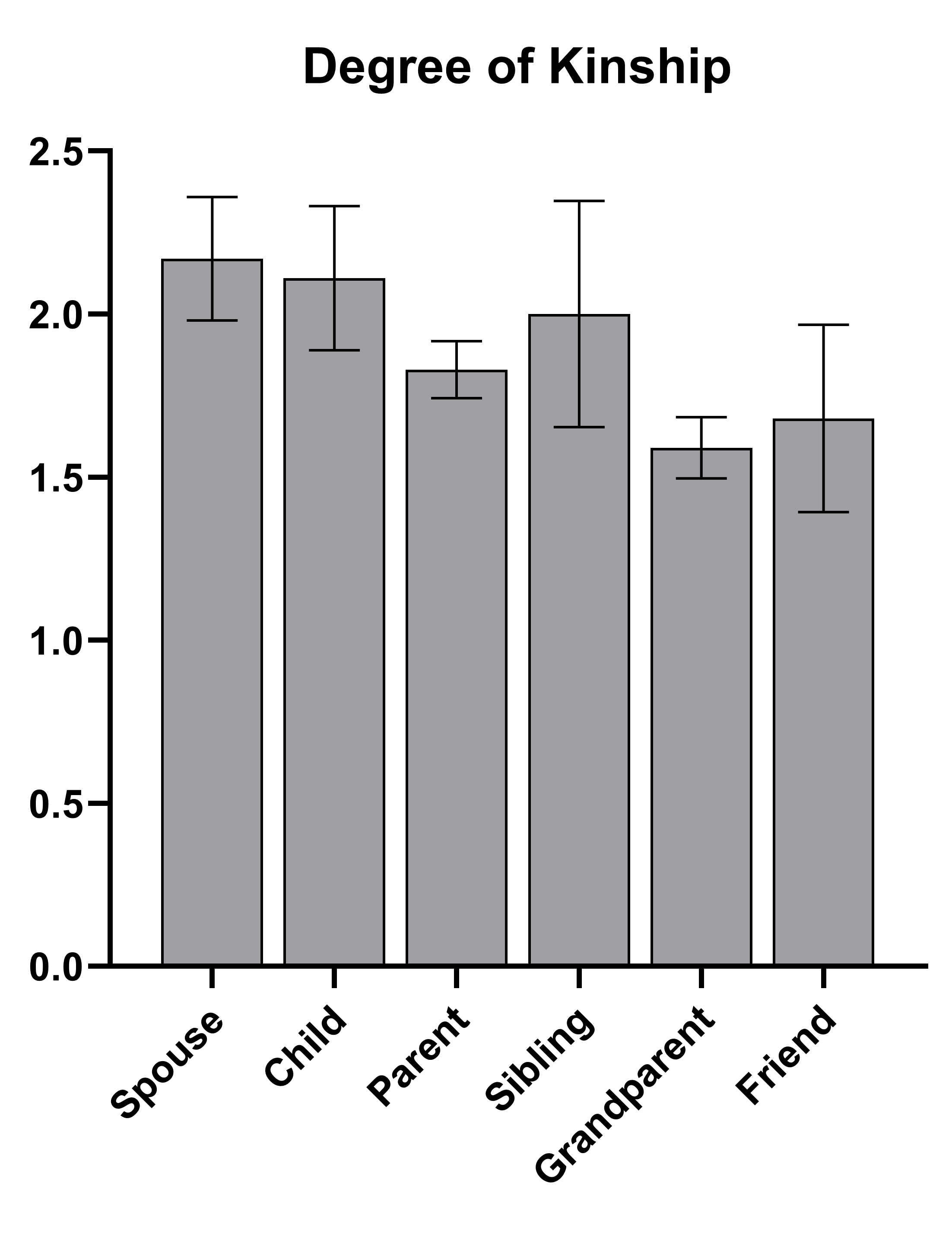

Supplement: S3 Fig — (TIF) [file pone.0320850.s006.tif]

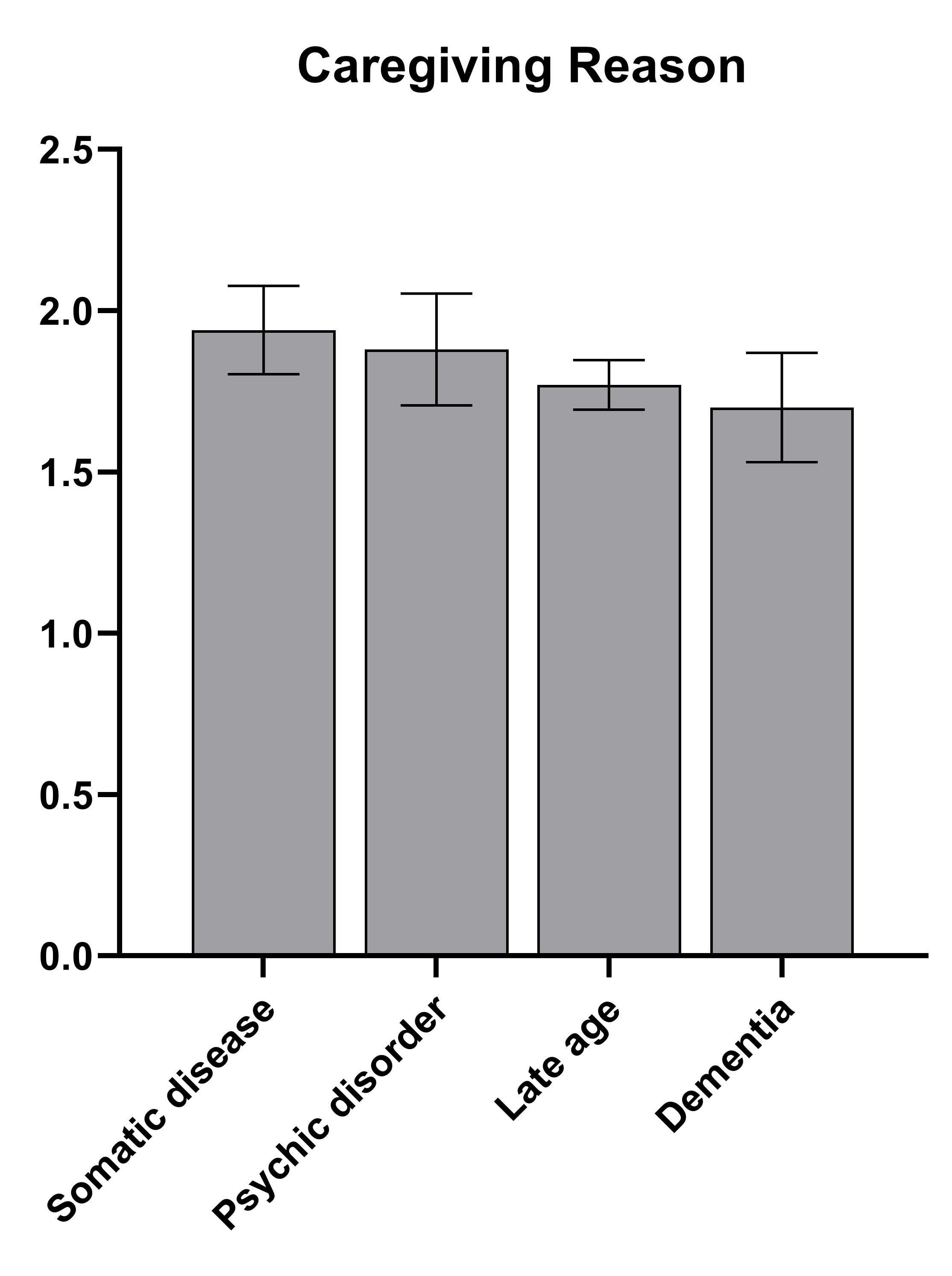

Supplement: S4 Fig — (TIF) [file pone.0320850.s007.tif]
