# Supplementary material for: Psychometric properties of the polish version of the Dysfunctional Thoughts about Caregiving Questionnaire (DTCQ)
Source: PLoS One. 2025 May 9;20(5):e0320850. doi: 10.1371/journal.pone.0320850 (PMC12063841; doi:10.1371/journal.pone.0320850)
Supplement: Attachment — Submitted filename: PONE-D-24-53758_reviewer.pdf [file pone.0320850.s008.pdf]

PLOS ONE

Psychometric Properties of the Polish Version of the Dysfunctional Thoughts About Caregiving Questionnaire

--Manuscript Draft--

**Manuscript Number:**	PONE-D-24-53758
**Article Type:**	Research Article
**Full Title:**	Psychometric Properties of the Polish Version of the Dysfunctional Thoughts About Caregiving Questionnaire
**Short Title:**	DTCQ - Psychometric Properties of the Polish Version
**Corresponding Author:**	Katarzyna Sanna, ph.d<br>SWPS University of Social Sciences and Humanities: SWPS Uniwersytet Humanistycznospoleczny<br>Poznań, POLAND
**Keywords:**	family caregiving; dysfunctional thoughts; Quality of Life; Measurement
**Abstract:**	Dysfunctional thoughts about caregiving are defined as rigid and inappropriate interpretations of one's experiences, behaviors, thoughts, or feelings that are associated with providing care to a dependent relative. The present study examined the psychometric properties of the Polish translation of the Dysfunctional Thoughts about Caregiving Questionnaire (DTCQ). DTCQ was administered to 618 family caregivers of people with chronic health problems. As the Confirmatory factor analysis supported both one and two-factor structure of the DTCQ, following the principle of parsimony, one-factor model was considered more appropriate. Construct validity was also supported by significant and theoretically expected relationships between DTCQ and depression, identity formation or sociodemographic variables. In conclusion, this study is the first to provide preliminary evidence for the reliability and validity of the DTCQ as a measure of dysfunctional thoughts in Polish family caregivers.
**Order of Authors:**	Katarzyna Maria Sanna, ph.d
	Maciej Kościelniak, Ph.d.
	Jarosław M Michałowski, Ph.d.
	Andrés Losada-Baltar, Ph.d.
	Konrad Piotrowski, Ph.d.
**Additional Information:**	
**Question**	**Response**
**Financial Disclosure**<br><br>Enter a financial disclosure statement that describes the sources of funding for the work included in this submission. Review the submission guidelines for detailed requirements. View published research articles from *PLOS ONE* for specific examples.<br><br>This statement is required for submission and **will appear in the published article** if the submission is accepted. Please make sure it is accurate.	Yes

Powered by Editorial Manager® and ProduXion Manager® from Aries Systems Corporation

**Funded studies**<br>Enter a statement with the following details:<br>• Initials of the authors who received each award<br>• Grant numbers awarded to each author<br>• The full name of each funder<br>• URL of each funder website<br>• Did the sponsors or funders play any role in the study design, data collection and analysis, decision to publish, or preparation of the manuscript?<br><br>Did you receive funding for this work?	
Please add funding details.<br>as follow-up to "**Financial Disclosure**<br><br>Enter a financial disclosure statement that describes the sources of funding for the work included in this submission. Review the submission guidelines for detailed requirements. View published research articles from *PLOS ONE* for specific examples.<br><br>This statement is required for submission and **will appear in the published article** if the submission is accepted. Please make sure it is accurate.<br><br>**Funded studies**<br>Enter a statement with the following details:<br>• Initials of the authors who received each award<br>• Grant numbers awarded to each author<br>• The full name of each funder<br>• URL of each funder website<br>• Did the sponsors or funders play any role in the study design, data collection and analysis, decision to publish, or preparation of the manuscript?<br><br>Did you receive funding for this work?"	The authors Katarzyna Sanna, Konrad Piotrowski, Maciej Kościelniak, Jarosław Michałowski received a research grant as part of the 6th call for grant proposals for financing research projects at the Institute of Psychology (6 konkurs na finansowanie projektów badawczych w Instytucie Psychologii), SWPS University, Warsaw, Poland. Grant number - 1268-11
Please select the country of your main research funder (please select carefully as in some cases this is used in fee calculation).	POLAND - PL

as follow-up to "**Financial Disclosure**<br><br>Enter a financial disclosure statement that describes the sources of funding for the work included in this submission. Review the submission guidelines for detailed requirements. View published research articles from *PLOS ONE* for specific examples.<br><br>This statement is required for submission and **will appear in the published article** if the submission is accepted. Please make sure it is accurate.<br><br>**Funded studies**<br>Enter a statement with the following details:<br>• Initials of the authors who received each award<br>• Grant numbers awarded to each author<br>• The full name of each funder<br>• URL of each funder website<br>• Did the sponsors or funders play any role in the study design, data collection and analysis, decision to publish, or preparation of the manuscript?<br><br>Did you receive funding for this work?"	
**Competing Interests**<br><br>Use the instructions below to enter a competing interest statement for this submission. On behalf of all authors, disclose any competing interests that could be perceived to bias this work—acknowledging all financial support and any other relevant financial or non-financial competing interests.<br><br>This statement is required for submission and **will appear in the published article** if the submission is accepted. Please make sure it is accurate and that any funding sources listed in your Funding Information later in the submission form are also declared in your Financial Disclosure statement.	The authors have declared that no competing interests exist

Powered by Editorial Manager® and ProduXion Manager® from Aries Systems Corporation

View published research articles from *PLOS ONE* for specific examples.<br><br>**NO authors have competing interests**<br><br>Enter: *The authors have declared that no competing interests exist.*<br><br>**Authors with competing interests**<br><br>Enter competing interest details beginning with this statement:<br><br>*I have read the journal's policy and the authors of this manuscript have the following competing interests: [insert competing interests here]*<br><br>* typeset	
**Ethics Statement**<br><br>Enter an ethics statement for this submission. This statement is required if the study involved:<br><br>• Human participants<br>• Human specimens or tissue<br>• Vertebrate animals or cephalopods<br>• Vertebrate embryos or tissues<br>• Field research<br><br>Write "N/A" if the submission does not require an ethics statement.<br><br>General guidance is provided below. Consult the submission guidelines for detailed instructions. **Make sure that all information entered here is included in the Methods section of the manuscript.**	This study received written approval from the Research Ethics Committee (Komisja Etyki) at SWPS University, Poznań, Poland (reference no. 2022-143)

**Format for specific study types**<br><br>**Human Subject Research (involving human participants and/or tissue)**<br>• Give the name of the institutional review board or ethics committee that approved the study<br>• Include the approval number and/or a statement indicating approval of this research<br>• Indicate the form of consent obtained (written/oral) or the reason that consent was not obtained (e.g. the data were analyzed anonymously)<br><br>**Animal Research (involving vertebrate animals, embryos or tissues)**<br>• Provide the name of the Institutional Animal Care and Use Committee (IACUC) or other relevant ethics board that reviewed the study protocol, and indicate whether they approved this research or granted a formal waiver of ethical approval<br>• Include an approval number if one was obtained<br>• If the study involved *non-human primates*, add *additional details* about animal welfare and steps taken to ameliorate suffering<br>• If anesthesia, euthanasia, or any kind of animal sacrifice is part of the study, include briefly which substances and/or methods were applied<br><br>**Field Research**<br><br>Include the following details if this study involves the collection of plant, animal, or other materials from a natural setting:<br>• Field permit number<br>• Name of the institution or relevant body that granted permission	
**Data Availability**<br><br>Authors are required to make all data underlying the findings described fully available, without restriction, and from the time of publication. PLOS allows rare exceptions to address legal and ethical concerns. See the PLOS Data Policy and FAQ for detailed information.	Yes - all data are fully available without restriction

A Data Availability Statement describing where the data can be found is required at submission. Your answers to this question constitute the Data Availability Statement and **will be published in the article**, if accepted.<br><br>**Important:** Stating 'data available on request from the author' is not sufficient. If your data are only available upon request, select 'No' for the first question and explain your exceptional situation in the text box.<br><br>Do the authors confirm that all data underlying the findings described in their manuscript are fully available without restriction?	
**Describe where the data may be found in full sentences. If you are copying our sample text, replace any instances of XXX with the appropriate details.**<br><br>• If the data are **held or will be held in a public repository**, include URLs, accession numbers or DOIs. If this information will only be available after acceptance, indicate this by ticking the box below. For example: *All XXX files are available from the XXX database (accession number(s) XXX, XXX.).*<br>• If the data are all contained **within the manuscript and/or Supporting Information files**, enter the following: *All relevant data are within the manuscript and its Supporting Information files.*<br>• If neither of these applies but you are able to provide **details of access elsewhere**, with or without limitations, please do so. For example:<br><br>*Data cannot be shared publicly because of [XXX]. Data are available from the XXX Institutional Data Access / Ethics Committee (contact via XXX) for researchers who meet the criteria for access to confidential data.*<br><br>*The data underlying the results presented in the study are available from (include the name of the third party*	All files will be available from the Open science framework. The URLs/accession numbers/DOIs will be available only after acceptance of the manuscript for publication.

Powered by Editorial Manager® and ProduXion Manager® from Aries Systems Corporation

and contact information or URL).<br>• This text is appropriate if the data are owned by a third party and authors do not have permission to share the data.<br><br>* typeset	
Additional data availability information:	Tick here if the URLs/accession numbers/DOIs will be available only after acceptance of the manuscript for publication so that we can ensure their inclusion before publication.

Manuscript Click here to access/download;Manuscript;DTCQ.docx

Psychometric Properties of the Polish Version of the Dysfunctional Thoughts About Caregiving Questionnaire

Katarzyna Sanna[1]¶*, Maciej Kościelniak[2] ¶, Jarosław M Michałowski[3]&, Andrés Losada-Baltar[4]&, Konrad Piotrowski[1]¶

[1]Center for Research on Personality Development, Institute of Psychology, SWPS University, Poznań, Poland

[2] Institute of Psychology, SWPS University, Poznań,Poland

[3]Poznan Laboratory of Affective Neuroscience, Institute of Psychology, SWPS University, Poznań,Poland

[4] Department of Psychology, Universidad Rey Juan Carlos, Madrid, Spain

* Corresponding author

ksanna@swps.edu.pl

¶ These authors contributed equally to this work.

& These authors also contributed equally to this work.

Abstract

Dysfunctional thoughts about caregiving are defined as rigid and inappropriate interpretations of one's experiences, behaviors, thoughts, or feelings that are associated with providing care to a dependent relative. The present study examined the psychometric properties of the Polish translation of the Dysfunctional Thoughts about Caregiving Questionnaire (DTCQ). DTCQ was administered to 618 family caregivers of people with chronic health problems. As the Confirmatory factor analysis supported both one and two-factor structure of the DTCQ, following the principle of parsimony, one-factor model was considered more appropriate. Construct validity was also supported by significant and theoretically expected relationships between DTCQ and depression, identity formation or sociodemographic variables. In conclusion, this study is the first to provide preliminary evidence for the reliability and validity of the DTCQ as a measure of dysfunctional thoughts in Polish family caregivers.

Introduction

As caregiving for a chronically ill person has proved to be a stressful and burdening experience for many family members, an increasing need for an in-depth look at the factors affecting the health and well-being of family caregivers has emerged [1-4]. In the literature, family members are called family caregivers, informal carers, family carers, or closest relatives who nurse their spouses, children, parents, distant relatives, or unrelated people [5]. Family caregivers perform a large number of tasks for many years and many hours per day that are associated with the illness, household duties, or emotional support of patients or other relatives [6-7]. As most family caregivers are not professionally prepared to perform specialized care-related activities, they are at risk of an increased sense of distress and burden, followed by other health problems [8-14]. Due to the challenges of an ageing society, increases in life expectancy even after being diagnosed with a life-threatening disease and the availability of institutional support, the adherence to personal values related to the importance of family (e.g., familism), and being cared for at home, family caregivers may remain the main source of care for many people [15]. Thus, the well-being of this population

should be a significant issue for healthcare providers and researchers studying the impact of health problems on family system functioning.

One of the factors related to the quality of life of family caregivers is their personal beliefs about 'good care' and their self-image as a caregiver. Unfortunately, the experience of caring for a dependent relative is often associated with the development and activation of many dysfunctional thoughts related to the care, which are defined as rigid and involve inappropriate interpretations of one's experiences, behaviors, thoughts, or feelings [16-17]. These types of thoughts that are learned through life experiences (e.g., influence of the family or the cultural con[illegible]) impose rigid, inflexible, and maladaptive rules of coping with caregiving (e.g., “A caregiver should only seek help from others or find other alternatives when the caregiving situation is at its worst or when they can no longer handle it”) and guide behavior (e.g., not seeking help and not devoting time to rest or leisure) [18]. These ways of thinking can act as cognitive barriers that make it difficult for the caregivers to take care of their own mental and physical health. Specifically, Montorio et al. [17] identified three types of interrelated dysfunctional beliefs connected to the caregiving experience. First, some of these beliefs may affect the amount of help that the caregivers receive for themselves. For instance, a caregiver may not seek any assistance from other family members or professionals as a result of a belief that a good caregiver should be able to handle all the caregiving tasks alone. Second, dysfunctional beliefs may create an excessive sense of perfectionism, responsibility, or excessive involvement in care, as the caregiver believes that only they are able to perform all care-related demands best. Third, this rigid way of thinking may prevent the expression of difficult emotions, as some caregivers believe they should not complain or verbalize any negative feelings. As a consequence, the caregiver does not allow themself to talk about the difficulties of care, which in the long run may result in a greater sense of burden. This way of thinking is highly dependent on cultural context, however. In particular, cultural values like familism are considered a factor that influences the way family members perceive their tasks regarding caregiving [20]. In cultures that cultivate family traditions, including Polish society [21], there is a cultural message that indicates

how caregivers should respond to the needs of dependent relatives, and that responsibility for care lies within the family [22]. According to the cognitive-behavioral model, these thoughts are responsible for the appearance of a negative mood, and in the long run for, among other effects, depression or anxiety [23-24].

Various dysfunctional thoughts-related questionnaires have been used in caregiving research. All of them were developed in the context of studies focused on family caregivers of people with dementia [19, 25-26]. One of the existing scales is the Dysfunctional Thoughts About Caregiving Questionnaire (DTCQ; [19]) —a self-report measure that assesses two different domains of caregiving [17]. The first domain refers to “perception of sole responsibility” in which the caregiver believes that a “good carer” should put care first and subordinate their own well-being and interests to the caregiving. The second domain refers to a sense of perfectionism, which represents high levels of emotional and behavioral self-demands. The questionnaire has high internal consistency and moderate test-retest reliability. The measure has Spanish, English, and French language versions [17, 19, 27], however the factor structure of the scale was verified only for the Spanish and English versions, showing the presence of two related factors.

Although the DTCQ was developed with a sample of family caregivers of people with dementia, the informal care experience affects family members of people facing a variety of conditions [28-29]. To more closely investigate this issue, in the present study we decided to use the DTCQ in a new area and assess its validity in a study of caregivers of people with a variety of health-related problems, including dementia, somatic diseases, mental health problems, or old-age frailty.

We specifically investigated the situation of family caregivers in Poland. Thus, our main objective was first to adapt the DTCQ [19], which would allow for the assessment of dysfunctional beliefs of family caregivers in Poland. Our second goal was to examine the relationships between the characteristics of family caregivers, contextual factors, and caregivers’ quality of life.

Studies on the consequences of informal caregiving have sought to identify factors that may affect care-related burden and negative caregiving outcomes (e.g., depressive symptoms; [30-31].

Some previous research indicated that the level of social support is one of the agents that affects adaptation to the informal caregiving experience [32], which can also be related to dysfunctional thoughts about caregiving. It was recently shown, however, that it is the support from family members rather than formal, institutional, or social support that can mitigate most of the negative consequences of caregiving [33]. In turn, the demographic variable that was previously considered as most related to caregivers' health was the gender of the caregiver, with women, compared to men, being more burdened with care [34]. However, recent research indicated that men also suffer negative consequences from providing care [35].

Another characteristic possibly related to adaptation to caregiving is the formation of and compliance with the caregivers' identity [36]. It is possible that caregivers' thoughts or beliefs may participate in the formation of identity. Studies based on processual identity models have confirmed that having a stable identity in various domains (e.g., romantic relationship, parenting, vocational activity) is one of the key predictors of adaptation, coping, and a high quality of life [37] . To our knowledge, there were currently no studies on the formation of caregivers' identity among family caregivers. However, research regarding parental identity (also related to the care of others) has shown that a stable sense of identity is associated with greater generativity and higher psychological and social well-being [38]. On the other hand, poor identification with the parental role has been associated with a high intensity of depressive symptoms, parental burnout, and a low quality of life [39]. In the present study, we hypothesized that the development of the carer's identity can also be linked to the level of their dysfunctional thoughts about caring.

The present study involved the Polish translation and validation of the DTCQ among a diverse population of family caregivers. We also aimed to verify whether the DTCQ used in more diverse groups of caregivers would retain the psychometric properties observed in samples of family caregivers of people with dementia. Specifically, we expected that the Polish version of the DTCQ would be characterized by a two-factor structure, similar to the original version, and would have acceptable internal consistency over .70 [40]. Additionally, we assumed that the correlation between

the two subscales (perception of sole responsibility and perfectionism) would be small to moderate, supporting two distinct aspects of dysfunctional beliefs related to caregiving. We also predicted that family caregivers' scores on the DTCQ dimensions would be related to the sense of identity in the caregiver role and several contextual factors that influence caregiving (e.g., kinship relationship, type of illness of the relative). Furthermore, we expected that DTCQ scores would positively correlate with depressive symptoms and negatively correlate with the social support the caregiver receives, and that higher levels of dysfunctional thoughts would be related to either a well-formed but rigid identity or to identity diffusion, manifested by a lack of acceptance of the caregiver role [39]. Regarding contextual factors, we predicted that the severity of dysfunctional thoughts would be positively related to the closeness of the relationship (i.e., the closer the relationship, the greater the sense of responsibility and perfectionism). As the DTCQ had been studied solely with dementia populations, we made no prior assumptions considering its relationships with the type of illness the close relative suffered from.

Materials and methods

Participants

The participants in this study were Polish family caregivers of people with chronic health problems. In order to participate in this study, respondents had to comply with the following inclusion criteria: caring for a sick family member/close relative and aged between 18 and 75 years. Before recruitment, we estimated the required sample size with G*Power 3.1.9.6 software [41]. For pairwise correlation analysis, assuming an effect size of .25, an alpha level of .05, and a power level of .95, the minimum required sample size was 202. For a one-way ANOVA, with the three comparison groups (effect size .25, alpha .05, power .95) that we planned to use, the minimum sample had to be 324. Because the structure of the DTCQ had not previously been studied in Poland, we assumed that the results might not support our hypotheses. In order to be able to carry out more

complex analyses in such a scenario, we considered recruiting a sample twice as large as the minimum.

Six hundred and eighteen caregivers of people chronically ill or requiring care participated in this study (*N* = 479; 77.5% female). The reasons for caregiving were (1) old-age frailty (*n* = 324; 52.5%), somatic disease (*n* = 146; 23.5%), a mental health disorder (*n* = 80; 13%), or dementia (*n* = 68; 11%). Most family caregivers cared for a parent (*n* = 271; 43.7%) or a grandparent (*n* = 182; 29.5%), followed by a spouse/wife (*n* = 58; 9.5%), child (*n* = 54; 8.7%), friend (*n* = 26; 4.2%), sibling (*n* = 25; 4%), or cousin (*n* = 2; 0.3%). The remaining sociodemographic characteristics of the sample are shown in Table 1.

Table 1

*The Descriptive Characteristics of 618 Family Caregivers in this Study's Sample (*N *= 618)*

Characteristic	
Gender (female %)	77.5%
Age	M = 42.2<br>SD = 14.84<br>Range = 18–75
Education (%)	Higher education: n = 304, 49.2%<br>Secondary education: n = 235, 38.2%<br>Primary education: n = 12, 1.9%<br>Vocational education: n = 61, 9.9%<br>Basic education: n = 5, 0.8%
Marital status (%)	Formal relationship: n = 300, 48.5%<br>Informal relationship: n = 175, 28.3%<br>Single: n = 143, 23.1%
Living area (%)	Countryside: n = 144, 23.3%<br>City > 10,000: n = 103, 16.7%<br>City > 100,000: n = 145, 23.5%<br>City > 500,000: n = 112, 18.1%<br>City < 500,000: n = 114, 18.4%
Kinship (per the person needing care) (%)	Spouse: n = 58, 9.4%<br>Parent: n = 271, 43.9%<br>Child: n = 54, 8.7%<br>Sibling: n = 25, 4%<br>Cousin: n = 2, 0.3%<br>Grandpa/grandma: n = 182, 29.4%

Characteristic	
Gender (female %)	77.5%
Age	M = 42.2<br>SD = 14.84<br>Range = 18–75
	Unrelated friend: n = 26, 4.2%
Caregiving reasons (%)	Somatic disease: n = 146, 23.6%<br>Mental health disorder: n = 80, 12.9%<br>Old-age frailty: n = 324, 52.4%<br>Dementia: n = 68, 11.1%
Level of the caregiver's perceived limitation of the caregiving (%)	Not at all: n = 21, 3.4%<br>To a small extent: n = 149, 24.1%<br>To a medium extent: n = 253, 40.9%<br>Mostly: n = 169, 27.3%<br>Entirely: n = 26, 4.2%
Financial situation (%)	Bad: n = 56, 9.1%<br>Average: n = 336, 54.4%<br>Good: n = 180, 29.1%<br>Very good: n = 46, 7.4%
Perceived health of the caregiver	Bad: n = 28, 4.5%<br>Average: n = 247, 40%<br>Good: n = 268, 43.4%<br>Very good: n = 75, 12.1%

Measures

The Dysfunctional Thoughts About Caregiving Questionnaire (DTCQ) The DTCQ [19] consists of 16 items and examines two dimensions: perception of sole responsibility (e.g., "*No matter how badly a caregiver feels, he/she should not vent with others because it would be disrespectful to the person being cared for*"), which means that the caregiver's priorities are the needs of the person under care, and perfectionism (e.g., "*A good caregiver should never get mad or lose control with the person that is being cared for*"), which represents a high level of caregiving demands in both emotional and behavioral aspects. All items were assessed on a 5-point Likert scale, from 0 (*I completely disagree*) to 4 (*I completely agree*). Information on the factor structure and reliability of the scale is given in the following section.

With the consent of the authors of the original DTCQ [19] , a standard back-translation was performed [42]. The questionnaire was translated into Polish by three independent translators. The fourth judge then compared the resulting versions, and the final Polish version of all items was determined. A native English speaker later translated the questionnaire back into English and compared it with the first author's original version. The Polish version prepared in this way was used in the present study. The psychometric properties of the Polish version are described in the results section below.

The Symptom Checklist-27-Plus (SCL-27-Plus) The SCL-27-plus [43] is a short, multidimensional screening instrument for mental health problems, containing five scales: depressive, vegetative, agoraphobic, social phobia, and pain symptoms. Each scale consists of between four and six items. The results are assessed based on the average of these items, with a higher score indicating a higher severity of symptoms of mental health problems. In this study, only a scale measuring the severity of depressive symptoms was used, consisting of five items, assessed based on their frequency of occurrence (the scale ranged from 1 (*never*) to 5 (*very often*). Cronbach's alpha was calculated, showing good reliability ($\alpha = 0.88$).

The Multidimensional Scale of Perceived Social Support (MSPSS) The MSPSS [44] is a self-report measure to assess perceived social support. It consists of 12 items with a 7-point Likert scale, from 1 (*I completely disagree*) to 7 (*I completely agree*). The MSPSS has a three-factor structure that refers to different sources of social support from family members, friends, or significant others. The results can be calculated both for individual subscales and for the entire scale. The higher the reported score, the higher the level of social support perceived by the person. Cronbach's alpha was calculated, showing excellent reliability ($\alpha = 0.93$).

The Utrecht-Management of Identity Commitments Scale, Informal Care (U-MICS-IC) The U-MICS [36, 45] measures the processes involved in identity development in different life domains. For this study, the scale was adapted for research with family caregivers (U-MICS-IC) by slightly modifying the items to relate to the care of a close adult. The scale consists of 13 items and assesses three

processes: (1) identity commitment (e.g., "*Being a caregiver [of this person] gives me a sense of security in life*"), (2) in-depth exploration (e.g., "*I often think about caring for [this person]*"), and (3) reconsideration of commitment (e.g., "*I often think that not having [that person] would make my life more interesting*"). A 5-point Likert scale is used to assess the individual items, with 1 (*completely false*) to 5 (*completely true*) answers. Since this was the first use of this version of the questionnaire, confirmatory factor analysis (CFA) was performed to assess its hypothesized three-factor structure. The analysis was performed with AMOS 29 [46}. CFA had shown that the three-factor solution fit the data well, with CFI = .95, SRMR = .05, and RMSEA = .07 [47]. Cronbach's alpha was calculated, showing acceptable reliability (α = 0.75).

Sociodemographic Characteristics A short sociodemographic questionnaire was created that included gender, marital status, level of education, reasons of caregiving, the level of perceived limitation from the caregiving, perceived health of the caregiver, and financial situation. Living area was measured on a 5-point scale, with a higher number indicating a locality with a greater number of inhabitants. The relationship with the person needing care was evaluated with a 7-point list of alternatives (i.e., "*he/she is my child,*" "*he/she is my parent*," "*he/she is my friend, we are not related*")

Ethical Considerations

This study received approval from the research ethics committee at the authors' university (reference no. 2022-143).

Procedure

This study was conducted with an online survey from 1st August 2022 to 22 November 2022. Information about the study was published on public social media and promoted by students at the authors' university. Written informed consent was obtained from all the participants enrolled in the research. The information included a statement about the purpose of the project and that participation was voluntary and free to terminate at any time; research specification including the number of questionnaires and time needed to complete them, the costs and benefits of participating

in the project, and the contact information of the main investigator in case of any questions. We excluded people who did not confirm that they were caring for a dependent person and people younger than 18 years and those older than 75 years. Once a participant selected all the required checkboxes, they were directed to the research survey questionnaires.

Data Analyses

We performed statistical analyses with JASP [48] (Version 0.14.1), SPSS (Version 29.0.2.0) [49], and Mplus [50]. In the first step, we conducted a normality analysis of all the studied continuous variables. To assess their univariate normality, we applied the cutoff criteria for skewness and kurtosis between -2 and +2 [51]. The distribution of the continuous variables turned out to be symmetrical. Apart from perceived social support (MSPSS), the distribution of all the continuous variables was symmetrical.

In order to analyze the factorial validity of the DTCQ, we compared one- and two-factor solutions [17, 19] with CFA. The analysis was performed with Mplus software [50], with the robust maximum likelihood (MLR) estimator. We applied several commonly used fit indices to evaluate and compare both solutions: comparative fit index (CFI), root mean square error of approximation (RMSEA), Tucker-Lewis index (TLI), and standardized root mean square residual (SRMR). We considered model fit to be adequate if the CFI and TLI values were higher than .90 and the RMSEA and SRMR values were lower than .08 [47]. Following the CFA, we assessed the reliability of the Polish DTCQ total score and subscale scores with McDonald's omega (ω). We considered a reliability coefficient of .70 or higher as acceptable.

Next, we calculated linear correlations between the DTCQ total score and its subdimensions with the criterion variables, using Pearson's *r* coefficient (for the MSPSS, we used Spearman's nonparametric test). Finally, we analyzed the associations between DTCQ scores and sociodemographic variables, including the caregiver's age, education level, and relationship/role with the person needing care.

Results

We conducted a CFA to evaluate the factor structure of the Polish adaptation of the DTCQ. Two models were tested: a one-factor model and a two-factor model with correlated factors (see Table 2 and Supplemental Materials). Both models demonstrated acceptable fit according to conventional criteria [47], with RMSEA values below .05; CFI and TLI values above .90; and SRMR values below .08. The χ^2/df ratios were all below 3, indicating an acceptable fit [52].

Table 2

Fit Indices for the DTCQ Models

Model	χ^2/df	RMSEA (90% CI)	CFI	TLI	SRMR
1. One-factor	2.45	.049 (.041–.056)	.948	.940	.036
2. Two correlated factors	2.41	.048 (.040–.055)	.950	.942	.036

Note. The Supplemental Materials contain detailed tables with model coefficients, factor loadings, and graphical representations of all tested models (i.e., one-factor, two-factor, and hierarchical).

When single-factor and more complex solutions in CFA demonstrate similar and acceptable goodness-of-fit parameters, the principle of parsimony suggests selecting the simpler, single-factor model [51]. This approach tends to yield more stable and replicable results across different samples, reducing the risk of overfitting and enhancing the model's generalizability [53]. Moreover, our CFA model revealed a much higher correlation between factors (r = .96) compared to the original paper (r = .65), which called into question the appropriateness of isolating two separate factors in this model, as postulated by the authors of this concept [17, 19]. Given these findings, we retained the one-factor model for the Polish adaptation of the DTCQ. This model provides a good balance between statistical fit and theoretical parsimony while avoiding the interpretational challenges posed by the high-factor correlation in the two-factor model.

Reliability

To assess the scale's internal consistency, Cronbach's alpha was calculated for the single-factor solution, with DTCQ scores computed as the mean value of 16 items, showing excellent reliability (α = 0.91).

External Validity of the DTCQ

To examine the relationships between dysfunctional thoughts about caregiving and various psychological and social factors, we conducted a correlational analysis, using the DTCQ general score. The results are presented in Table 3.

Table 3

Correlations of the DTCQ General Score With External Measures

Variable	*r*	*p*
SCL-27 Depression	.12	.003
MSPSS Total	-.01	.310
MSPSS Family	.03	.476
MSPSS Friends	-.04	.350
MSPSS Significant Others	-.01	.902
U-MICS Commitment	.24	<.001
U-MICS In-Depth Exploration	.09	.023
U-MICS Reconsideration of Commitment	-.04	.308

Note. *p < .05, **p < .01, ***p < .001. SCL-27 = Symptom Checklist-27+ Questionnaire; MSPSS = Multidimensional Scale of Perceived Social Support; U-MICS = Utrecht-Management of Identity Commitments Scale.

The analysis revealed a significant positive correlation between the DTCQ score and depressive symptoms. This suggests that higher levels of dysfunctional thoughts about caregiving were associated with more depressive symptomatology.

Regarding social support, as measured by the MSPSS, no significant correlations were found between the DTCQ score and overall perceived social support or support from specific sources (family, friends, and significant others). This indicates that dysfunctional thoughts about caregiving were not necessarily related to perceived social support in our sample. We also found significant

positive correlations between the DTCQ score and both commitment and in-depth exploration, suggesting interdependencies between levels of dysfunctional thoughts about caregiving and identity commitment and in-depth exploration.

Sociodemographic Aspects of Dysfunctional Thoughts About Caregiving

Gender and Age An independent samples *t*-test revealed a significant difference in DTCQ scores between genders (Fig 1), $t(616) = 3.89$, $p < .001$, $d = 0.375$. Men ($M = 2.03$, $SD = 0.672$) scored significantly higher than women ($M = 1.76$, $SD = 0.745$). Age was positively correlated with DTCQ scores, $r(616) = .223$, $p < .001$, indicating that older caregivers tended to report higher levels of dysfunctional thoughts about caregiving.

Fig 1 Mean DTCQ Scores by Gender. Note. Error bars represent 95% confidence intervals of the mean

Education A one-way ANOVA showed a significant effect of education on DTCQ scores, $F(3, 614) = 10.4$, $p < .001$, $\eta^2p = .048$ (Fig 2). Post-hoc comparisons with Bonferroni correction revealed that individuals with higher education scored significantly lower on the DTCQ compared to those with vocational education ($p < .001$) and secondary education ($p < .001$).

Fig 2 Mean DTCQ Scores by Education. Note. Error bars represent 95% confidence intervals of the mean

Degree of Kinship With the Patient We observed a significant effect of the degree of kinship on DTCQ scores, $F(5, 610) = 8.89$, $p < .001$, $\eta^2p = .068$ (Fig 3). Post-hoc tests showed that caregivers of grandparents scored significantly lower compared to spouses ($p < .001$) and children ($p < .001$) of patients. Additionally, caregivers of parents scored lower than spouses ($p = .013$).

Fig 3 Mean DTCQ Scores by Degree of Kinship with the Patient. Note. Error bars represent 95% confidence intervals of the mean

Caregiving Reason The reason for caregiving had a significant effect on DTCQ scores, $F(3, 614) = 2.74$, $p = .042$, $\eta^2p = .013$ (Fig 4). However, post-hoc comparisons did not reveal significant pairwise

differences after Bonferroni correction. A trend suggested that caregivers of individuals with somatic diseases scored higher on the DTCQ than those caring for people due to old-age frailty (p = .090).

Fig 4 Mean DTCQ Scores by Caregiving Reasons. Note. Error bars represent 95% confidence intervals of the mean

Discussion

In the present study we evaluated the psychometric properties of the Polish version of the DTCQ and the links of dysfunctional thoughts with other caregiver characteristics. The obtained results per factor structure, internal consistency, and convergent validity suggest the DTCQ can be considered a valuable measure to assess dysfunctional thoughts among family caregivers of people with diverse chronic illnesses.

CFA indicated that both a one-factor and two-factor structure of the DTCQ fit the data well. Although the authors of the original version have suggested a two-factor solution, our results show that in Polish caregivers, a one-factor model is more suitable, reflecting the existence of a global predisposition towards dysfunctional thinking about caregiving. While the original study and its French version [17, 19, 27] found enough distinction to justify a two-factor structure, our results indicate that in the Polish cultural context, the perfectionism and responsibility dimensions were so highly interrelated that they may be better represented as a single, overarching construct. This difference might reflect cultural variations in how caregiving responsibilities and expectations are perceived and experienced in Poland compared to the original study's context, highlighting the importance of cultural adaptation in psychological measurement. The findings also indicate that the measure can be used with more diverse groups while maintaining its psychometric properties. This notion seems to be of great importance since the caregiving experience applies to people caring for those who need care for various reasons [54- 55], and not only for people with dementia.

Our findings revealed positive relationships between DTCQ scores with identity commitment, showing that dysfunctional beliefs about caregiving are associated with a firm, stable

identity, possibly also with a rigid self-image as a caregiver [17]. The observed positive correlations with in-depth exploration indicate that dysfunctional beliefs may co-occur with greater motivation to seek in-depth information about caregiving. Interestingly, no correlations were observed with reconsideration of commitment—an indicator of non-acceptance of the caregiver role. It appears that dysfunctional thoughts related to caregiving co-occurred with a strong, rigid, and well-defined caregiver identity rather than a diffuse identity and lack of acceptance of the caregiver role. This relationship can go in two different directions: It can be predicted that the dysfunctional thoughts associated with caregiving, such as the belief that only the individual can handle the task and that the carer should always be ready to care, support the emergence of such a well-defined identity, or on the other hand, a strong role identification may be a risk factor for experiencing care-related dysfunctional thoughts. Dysfunctional thoughts could have a double-edged sword effect, first helping to identify with the caregiving role and focus on caregiving tasks, but with long-term negative effects, by favoring maladaptive coping strategies such as reducing leisure time or inspiring less help-seeking [56]. Future research in this domain is needed to assess how dysfunctional thoughts may hinder or facilitate the process of adaptation and subsequent fulfilment of the role of a caregiver, and longitudinal studies might identify causal relationships between dysfunctional thoughts and caregiver identity development.

As expected, the DTCQ was linked to depressive symptoms. Our findings are mostly consistent with those of other studies (e.g. [17]) and the cognitive model [57-58], suggesting that dysfunctional thoughts are responsible for the development of depressive symptoms. The more rigid and self-demanding the perception of caregiving, the more strain and burden a caregiver may experience, leading to the feeling of overwhelm, a lack of hope and resources, and then eventually depression. Conversely, the severity of depressive symptoms may increase the intensity and frequency of dysfunctional thoughts [23], resulting in a greater risk of experiencing care-related negative beliefs due to a depressed mood before starting care.

Contrary to our expectations, we did not find links between the DTCQ and social support, similar to results obtained in previous studies [23]. Nevertheless, apart from the MSPSS subscale Family, the direction of these relationships remained consistent with the results of other studies [17, 59]. It is also possible that in our Polish caregiver sample, factors other than social support were more related to dysfunctional thinking, such as the use of formal support resources (e.g., home care or day care). Overall, these findings provide insights into the complex relationships between dysfunctional thoughts about caregiving and various psychological and social factors, highlighting potential areas for further research and caregiver support interventions.

Regarding the sociodemographic characteristics, the proportion of female and male caregivers in our study confirms the results of previous studies indicating that women are more likely to take on the role of caregivers [59]. In contrast to our expectations, but similar to the results obtained by Montorio et al. [17] , men reported more dysfunctional thoughts compared to women. This result confirms the latest research findings, indicating that both women and men experience negative consequences from caregiving, but perhaps in a different way [3, 60]. Also, the differential effects between age and education level have precedents in other family care research [17, 61]. It appears that older age and lower levels of education are risk factors for experiencing dysfunctional thoughts about caregiving.

Additionally, on closer examination, very interesting information came from comparing the intensity of dysfunctional thoughts between people who had different relationships with the patient. It appeared that the closer the social relationship was (e.g., child, spouse), the more dysfunctional thoughts about caregiving were present. These results are consistent with the results of another study that found that the severity of dysfunctional thoughts and psychopathological symptoms may depend on the degree of kinship and gender [52].

Finally, the results of our study suggest that the diagnosis of the family member is secondary to the severity of dysfunctional thoughts. Tandentik et al. [27] found differences in the intensity of dysfunctional thoughts between caregivers of patients with Alzheimer's disease and frontotemporal

dementia. Nevertheless, these are disorders from the neurodegenerative spectrum, and to our knowledge, studies with more diverse groups have not been conducted. Therefore, perhaps it is not the disorder itself that is responsible for the negative experience of care but the coincidence of the cognitive profile and available resources in a cultural context that may be key for understanding the development and impact of dysfunctional thoughts [56, 63]

Study Limitations

This study had some limitations. First, although we found no significant differences between the family member’s diagnosis and dysfunctional thoughts, our sample was half-represented by caregivers of older people. Also, people with lower education were underrepresented, and the study was conducted online, thus the research was limited to internet users, and it is impossible to generalize these findings to general caregiving populations. Considering that our population of caregivers consisted also of older people that may not have had access to a computer, future research might replicate these findings with a sample of Polish caregivers recruited through other means than online research. Future studies should seek more representative samples per educational levels regarding the caregiver sample and also include caregivers of people with somatic and mental health disorders in order to paint a fuller picture.

Another limitation pertained to the data collection method. We used self-report questionnaires that did not include scales measuring potential sources of bias in the answers provided (e.g., social desirability; [59]). Due to the sensitive topic of our study and perhaps the respondents' desire to appear in a better light, the results obtained may not fully reflect the severity of the variables. We recommend introducing scales that measure these features in future research.

Finally, we did not measure the convergent validity of the DTCQ with other measures of dysfunctional thoughts, as in Poland there is no alternative DTCQ questionnaire. Should an adaptation of another measure be published, it would be desirable to carry out such an analysis.

Conclusion

The presented results suggest that the Polish version of the DTCQ is a reliable and valid measure for the assessment of caregivers' specific dysfunctional attitudes in various groups of caregivers. Considering an ageing society with a longer life expectancy and higher risks of health-related need for care, a tool like this might be very helpful for identifying people who find it difficult to adapt to the role of caregiver and to introduce adequate interventions for preventing or targeting psychological distress and other care-related vulnerabilities.

References

1. Ehsan N, Johar N, Saleem T, Khan MA, Ghauri S. Negative repercussions of caregiving burden: Poor psychological well-being and depression. Pakistan Journal of Medical Sciences [Internet]. 2018 Nov 1 [cited 2020 Oct 26];34(6):1452–6.
2. Roth DL, Fredman L, Haley WE. Informal caregiving and its impact on health: a reappraisal from population-based studies. Gerontologist [Internet]. 2015;55(2):309–19. Available from: http://dx.doi.org/10.1093/geront/gnu177
3. Verbakel E, Tamlagsrønning S, Winstone L, Fjær EL, Eikemo TA. Informal care in Europe: findings from the European Social Survey (2014) special module on the social determinants of health. Eur J Public Health [Internet]. 2017;27(suppl_1):90–5. Available from: http://dx.doi.org/10.1093/eurpub/ckw229
4. Zwar L, König H-H, Hajek A. Psychosocial consequences of transitioning into informal caregiving in male and female caregivers: Findings from a population-based panel study. Soc Sci Med [Internet]. 2020;264(113281):113281. Available from: http://dx.doi.org/10.1016/j.socscimed.2020.113281
5. Sanna K. Application and effectiveness of Cognitive Behavioural Therapy among informal caregivers of chronically ill people- literature review. Psychoterapia [Internet]. 2022;199(4):65–74. Available from: http://dx.doi.org/10.12740/pt/146397

6. Andersen HE, Hoeck B, Nielsen DS, Ryg J, Delmar C. A phenomenological-hermeneutic study exploring caring responsibility for a chronically ill, older parent with frailty. Nurs Open [Internet]. 2020;7(4):951–60. Available from: http://dx.doi.org/10.1002/nop2.467
7. Teixeira RJ, Applebaum AJ, Bhatia S, Brandão T. The impact of coping strategies of cancer caregivers on psychophysiological outcomes: an integrative review. Psychol Res Behav Manag [Internet]. 2018;11:207–15. Available from: http://dx.doi.org/10.2147/prbm.s164946
8. Abreu W, Tolson D, Jackson GA, Costa N. A cross-sectional study of family caregiver burden and psychological distress linked to frailty and functional dependency of a relative with advanced dementia. Dementia [Internet]. 2020;19(2):301–18. Available from: http://dx.doi.org/10.1177/1471301218773842
9. Barrera-Caballero S, Romero-Moreno R, Del Sequeros Pedroso-Chaparro M, Olmos R, Vara-García C, Gallego-Alberto L, et al. Stress, cognitive fusion and comorbid depressive and anxiety symptomatology in dementia caregivers. Psychol Aging [Internet]. 2021;36(5):667–76. Available from: http://dx.doi.org/10.1037/pag0000624
10. Del-Pino-Casado R, Priego-Cubero E, López-Martínez C, Orgeta V. Subjective caregiver burden and anxiety in informal caregivers: A systematic review and meta-analysis. PLoS One [Internet]. 2021;16(3):e0247143. Available from: http://dx.doi.org/10.1371/journal.pone.0247143
11. Geng H-M, Chuang D-M, Yang F, Yang Y, Liu W-M, Liu L-H, et al. Prevalence and determinants of depression in caregivers of cancer patients: A systematic review and meta-analysis. Medicine (Baltimore) [Internet]. 2018;97(39):e11863. Available from: http://dx.doi.org/10.1097/md.0000000000011863
12. Kaddour L, Kishita N. Anxiety in informal dementia carers: A meta-analysis of prevalence. J Geriatr Psychiatry Neurol [Internet]. 2020;33(3):161–72. Available from: http://dx.doi.org/10.1177/0891988719868313
13. Koyanagi A, DeVylder JE, Stubbs B, Carvalho AF, Veronese N, Haro JM, et al. Depression,

sleep problems, and perceived stress among informal caregivers in 58 low-, middle-, and high-income countries: A cross-sectional analysis of community-based surveys. J Psychiatr Res [Internet]. 2018;96:115–23. Available from: http://dx.doi.org/10.1016/j.jpsychires.2017.10.001

14. Loh AZ, Tan JS, Zhang MW, Ho RC. The global prevalence of anxiety and depressive symptoms among caregivers of stroke survivors. J Am Med Dir Assoc [Internet]. 2017;18(2):111–6. Available from: http://dx.doi.org/10.1016/j.jamda.2016.08.014
15. Ambugo EA, de Bruin SR, Masana L, MacInnes J, Mateu NC, Hagen TP, et al. A cross-European study of informal carers' needs in the context of caring for older people, and their experiences with professionals working in integrated care settings. Int J Integr Care [Internet]. 2021;21(3):2. Available from: http://dx.doi.org/10.5334/ijic.5547
16. Beck J. Terapia poznawczo-behawioralna. Kraków: Wydawnictwo Uniwersytetu Jagiellońskiego; 2012.
17. Montorio I, Losada A, Izal M, Márquez M. Dysfunctional thoughts about caregiving questionnaire: psychometric properties of a new measure. Int Psychogeriatr [Internet]. 2009;21(5):913–21. Available from: http://dx.doi.org/10.1017/S1041610209990366
18. Losada A, Montorio I, Knight BG, Márquez M, Izal M. Explanation of caregivers distress from the cognitive model: the role of dysfunctional thoughts. Psicol Conductual. 2006; 14: 115-128.
19. Losada A, Montorio I, Izal M, Márquez-González M. Estudio e intervención sobre el malestar psicológico de los cuidadores de personas con demencia: El papel de los pensamientos disfuncionales. Study and intervention on dementia caregiving distress: The role of dysfunctional thoughts. Madrid: IMSERSO; 2006.
20. Cabrera I, Márquez-González M, Kishita N, Vara-García C, Losada A. Development and validation of an Implicit Relational Assessment Procedure (IRAP) to measure implicit dysfunctional beliefs about caregiving in dementia family caregivers. Psychol Rec [Internet].

2021;71(1):41–54. Available from: http://dx.doi.org/10.1007/s40732-020-00445-8

21. Swadźba U. (2015). Różnice i podobieństwa Miejsce religii i rodziny w systemie wartości społeczeństwa polskiego, czeskiego i słowackiego. Górnośląskie Studia Socjologiczne. Seria Nowa 6. 2015; 101-122.
22. Losada A, Marquez-Gonzalez M, Knight BG, Yanguas J, Sayegh P, Romero-Moreno R. Psychosocial factors and caregivers' distress: effects of familism and dysfunctional thoughts. Aging Ment Health [Internet]. 2010;14(2):193–202. Available from: http://dx.doi.org/10.1080/13607860903167838
23. Leahy RL, Holland SJF. Treatment plans and interventions for depression and anxiety disorders. New York, NY: Guilford Publications; 2011.
24. Yapan S, Türkçapar, MH, Boysan M. Rumination, automatic thoughts, dysfunctional attitudes, and thought suppression as transdiagnostic factors in depression and anxiety. Curr Psychol [Internet] 2020;41(9): 5896–5912. Available from: https://doi.org/10.1007/s12144-020-01086-4
25. Risch AK, Mund M, Wilz G. The caregiver thoughts scale: An instrument to assess functional and dysfunctional thoughts about caregiving. Clin Gerontol [Internet]. 2023;46(4):585–98. Available from: http://dx.doi.org/10.1080/07317115.2022.2153775
26. Sullivan KA, Beattie E, Khawaja NG, Wilz G, Cunningham L. . The thoughts questionnaire (TQ) for family caregivers of people with dementia. Dementia [Internet]. 2016;15(6): 1474–1493. Available from: https://doi.org/10.1177/1471301214553038
27. Tandetnik C, Hergueta T, Negovanska V, Dubois B, Bungener C. Traduction française du « Dysfunctional Thoughts about Caregiving Questionnaire- DTCQ ». Étude préliminaire auprès d' aidants de personnes atteintes de maladies neurodégénératives. Journal de thérapie comportementale et cognitive. 2014;24: 5-13. .
28. Kim B, Wister A, O'dea E, Mitchell BA, Li L, Kadowaki L. Roles and experiences of informal caregivers of older adults in community and healthcare system navigation: a scoping review.

BMJ Open [Internet]. 2023;13(12):e077641. Available from: http://dx.doi.org/10.1136/bmjopen-2023-077641

29. Molassiotis A, Wang M. Understanding and supporting informal cancer caregivers. Curr Treat Options Oncol [Internet]. 2022;23(4):494–513. Available from: http://dx.doi.org/10.1007/s11864-022-00955-3
30. Cejalvo E, Martí-Vilar M, Merino-Soto C, Aguirre-Morales MT. Caregiving role and psychosocial and individual factors: A systematic review. Healthcare (Basel) [Internet]. 2021;9(12):1690. Available from: http://dx.doi.org/10.3390/healthcare9121690
31. Stratmann M, Forsell Y, Möller J, Liang Y. Informal care and the impact on depression and anxiety among Swedish adults: a population-based cohort study. BMC Public Health [Internet]. 2021;21(1):1263. Available from: http://dx.doi.org/10.1186/s12889-021-11246-1
32. Díaz M, Estévez A, Momeñe J, Ozerinjauregi N. Social support in the relationship between perceived informal caregiver burden and general health of female caregivers. Ansiedad estrés [Internet]. 2019;25(1):20–7. Available from: http://dx.doi.org/10.1016/j.anyes.2019.04.001
33. Shiba K, Kondo N, Kondo K. Informal and formal social support and caregiver burden: The AGES caregiver survey. J Epidemiol [Internet]. 2016;26(12):622–8. Available from: http://dx.doi.org/10.2188/jea.JE20150263
34. del-Pino-Casado R, Frías-Osuna A, Palomino-Moral PA, Ramón Martínez-Riera J. Gender differences regarding informal caregivers of older people: Gender differences in caregiving. J Nurs Scholarsh [Internet]. 2012;44(4):349–57. Available from: http://dx.doi.org/10.1111/j.1547-5069.2012.01477.x
35. Bhan N, Rao N, Raj A. Gender differences in the associations between informal caregiving and wellbeing in low- and middle-income countries. J Womens Health (Larchmt) [Internet]. 2020;29(10):1328–38. Available from: http://dx.doi.org/10.1089/jwh.2019.7769
36. Crocetti E, Rubini M, Meeus W. Capturing the dynamics of identity formation in various

ethnic groups: development and validation of a three-dimensional model. J Adolesc [Internet]. 2008;31(2):207–22. Available from: http://dx.doi.org/10.1016/j.adolescence.2007.09.002

37. Crocetti E, Meeus W. The identity statuses: Strengths of a person-centered approach. In: McLean KC, Syed M, editors. The Oxford handbook of identity development. Oxford: Oxford University Press; 2015. p.97-114.
38. Fadjukoff P, Pulkkinen L, Lyyra A-L, Kokko K. Parental identity and its relation to parenting and psychological functioning in middle age. Parent Sci Pract [Internet]. 2016;16(2):87–107. Available from: http://dx.doi.org/10.1080/15295192.2016.1134989
39. Piotrowski K. How many parents regret having children and how it is linked to their personality and health: Two studies with national samples in Poland. PLoS One [Internet]. 2021;16(7):e0254163. Available from: http://dx.doi.org/10.1371/journal.pone.0254163
40. Cortina JM. What is coefficient alpha? An examination of theory and applications. J Appl Psychol [Internet]. 1993;78(1):98–104. Available from: http://dx.doi.org/10.1037/0021-9010.78.1.98
41. Faul F, Erdfelder E, Lang A-G, Buchner A. G*Power 3: a flexible statistical power analysis program for the social, behavioral, and biomedical sciences. Behav Res Methods [Internet]. 2007;39(2):175–91. Available from: http://dx.doi.org/10.3758/bf03193146
42. Brislin RW. The wording and translation of research instruments. In: Lonner WJ, Berry JW, editors. Field methods in cross-cultural research. Newbury Park, CA: Sage; 1986. p. 137-164.
43. Kuncewicz D, Dragan M, Hardt J. (2014). Walidacja polskiej wersji kwestionariusza The Symptom Checklist-27-plus. Psychiatria Polska. 2014;48(2): 345-358.
44. Buszman K. Przybyła - Basista H. (2017). Polska adaptacja wielowymiarowej skali spostrzeganego wsparcia społecznego. Polskie Forum Psychologiczne. 2017;22(4):581-599.
45. Piotrowski K. Adaptation of the Utrecht-Management of Identity Commitments Scale (U-MICS) to the measurement of the parental identity domain. Scand J Psychol [Internet].

2018;59(2):157–66. Available from: http://dx.doi.org/10.1111/sjop.12416

46. Arbuckle JL. Amos. [Computer Program] (Version 29.0) Chicago: IBM SPSS. 2022.

47. Hu L-T, Bentler PM. Cutoff criteria for fit indexes in covariance structure analysis: Conventional criteria versus new alternatives. Struct Equ Modeling [Internet]. 1999;6(1):1–55. Available from: http://dx.doi.org/10.1080/10705519909540118

48. JASP Team. JASP. [Computer Software] (version 0.14.1).2014.

49. IBM Corporation. IBM SPSS Statistics for Macintosh. [Computer Program] (version 29.0.2.0) 2023.

50. Muthén LK, Muthén BO. Mplus User's Guide. Eighth Edition. Los Angeles, CA: Muthén & Muthén; 1998-2023.

51. Curtin F, Schulz P. Multiple correlations and bonferroni’s correction. Biol Psychiatry [Internet]. 1998;44(8):775–7. Available from: http://dx.doi.org/10.1016/s0006-3223(98)00043-2

52. Kline RB. Principles and practice of structural equation modeling. New York, NY;Guilford publications; 2015.

53. Brown TA. Confirmatory factor analysis for applied research, second edition. 2nd ed. New York, NY: Guilford Publications; 2015.

54. Kent EE, Mollica MA, Buckenmaier S, Wilder Smith A. The characteristics of informal cancer caregivers in the United States. Semin Oncol Nurs [Internet]. 2019;35(4):328–32. Available from: http://dx.doi.org/10.1016/j.soncn.2019.06.002

55. Tur-Sinai A, Teti A, Rommel A, Hlebec V, Lamura G. How many older informal caregivers are there in Europe? Comparison of estimates of their prevalence from three European surveys. Int J Environ Res Public Health [Internet]. 2020;17(24):9531. Available from: http://dx.doi.org/10.3390/ijerph17249531

56. Huertas-Domingo C, Márquez-González M, Cabrera I, Barrera-Caballero S, Pedroso-Chaparro MDS, Romero-Moreno R, et al. Sociocultural influences on the feeling of loneliness of family

caregivers of people with dementia: The role of kinship. Int J Environ Res Public Health [Internet]. 2021;18(9):4700. Available from: http://dx.doi.org/10.3390/ijerph18094700

57. Barrera-Caballero S, Romero-Moreno R, Márquez-González M, Jiménez-Gonzalo L, Huertas-Domingo C, Olazarán J, et al. Longitudinal effects of cognitive fusion in depressive and anxious symptoms of family caregivers of people with dementia. J Contextual Behav Sci [Internet]. 2024;33(100782):100782. Available from: http://dx.doi.org/10.1016/j.jcbs.2024.100782

58. Beck AT, Dozois DJA. Cognitive therapy: current status and future directions. Annu Rev Med [Internet]. 2011;62(1):397–409. Available from: http://dx.doi.org/10.1146/annurev-med-052209-100032

59. Pacheco Barzallo D, Schnyder A, Zanini C, Gemperli A. Gender Differences in Family Caregiving. Do female caregivers do more or undertake different tasks? BMC Health Serv Res [Internet]. 2024;24(1):730. Available from: http://dx.doi.org/10.1186/s12913-024-11191-w

60. Xiong C, Biscardi M, Nalder E, Colantonio A. Sex and gender differences in caregiving burden experienced by family caregivers of persons with dementia: a systematic review protocol. BMJ Open [Internet]. 2018;8(8):e022779. Available from: http://dx.doi.org/10.1136/bmjopen-2018-022779

61. Schnitzer S, Oedekoven M, Amin-Kotb K, Gellert P, Balke K, Kuhlmey A. Caregivers' burden and education level: Does subjective health mediate the association? Innov Aging [Internet]. 2017;1(suppl_1):447–447. Available from: http://dx.doi.org/10.1093/geroni/igx004.1599

62. Huertas-Domingo C, Losada-Baltar A, Gallego-Alberto L, García-Batalloso I, García-García L, Márquez-González M. Profiles of dementia caregivers according to psychosocial and resource variables. Importance of kinship. Span J Psychol [Internet]. 2024;27(e15):e15. Available from: http://dx.doi.org/10.1017/SJP.2024.15

63. Knight BG, Sayegh P. Cultural values and caregiving: the updated sociocultural stress and coping model. J Gerontol B Psychol Sci Soc Sci [Internet]. 2010;65B(1):5–13. Available from:

http://dx.doi.org/10.1093/geronb/gbp096

Supporting information

S1 Fig 1 Mean DTCQ Scores by Gender**.** Note. Error bars represent 95% confidence intervals of the mean

S2 Fig 2 Mean DTCQ Scores by Education**.** Note. Error bars represent 95% confidence intervals of the mean

S3 Fig 3 Mean DTCQ Scores by Degree of Kinship with the Patient. Note. Error bars represent 95% confidence intervals of the mean

S4 Fig 4 Mean DTCQ Scores by Caregiving Reasons Note. Error bars represent 95% confidence intervals of the mean

S1 Table 1. The Descriptive Characteristics of 618 Family Caregivers in this Study's Sample (N = 618)**.**

S2 Table 2. Fit Indices for the DTCQ Models**.** Note. The Supplemental Materials contain detailed tables with model coefficients, factor loadings, and graphical representations of all tested models (i.e., one-factor, two-factor, and hierarchical).

S3 Table 3. *Correlations of the DTCQ General Score With External Measures. Note.* **p* < .05, ***p* < .01, ****p* < .001. SCL-27 = Symptom Checklist-27+ Questionnaire; MSPSS = Multidimensional Scale of Perceived Social Support; U-MICS = Utrecht-Management of Identity Commitments Scale.

S1 File 1. Supplementary Materials - DTCQ

Figure

Click here to access/download;Figure;S1_Fig..tiff

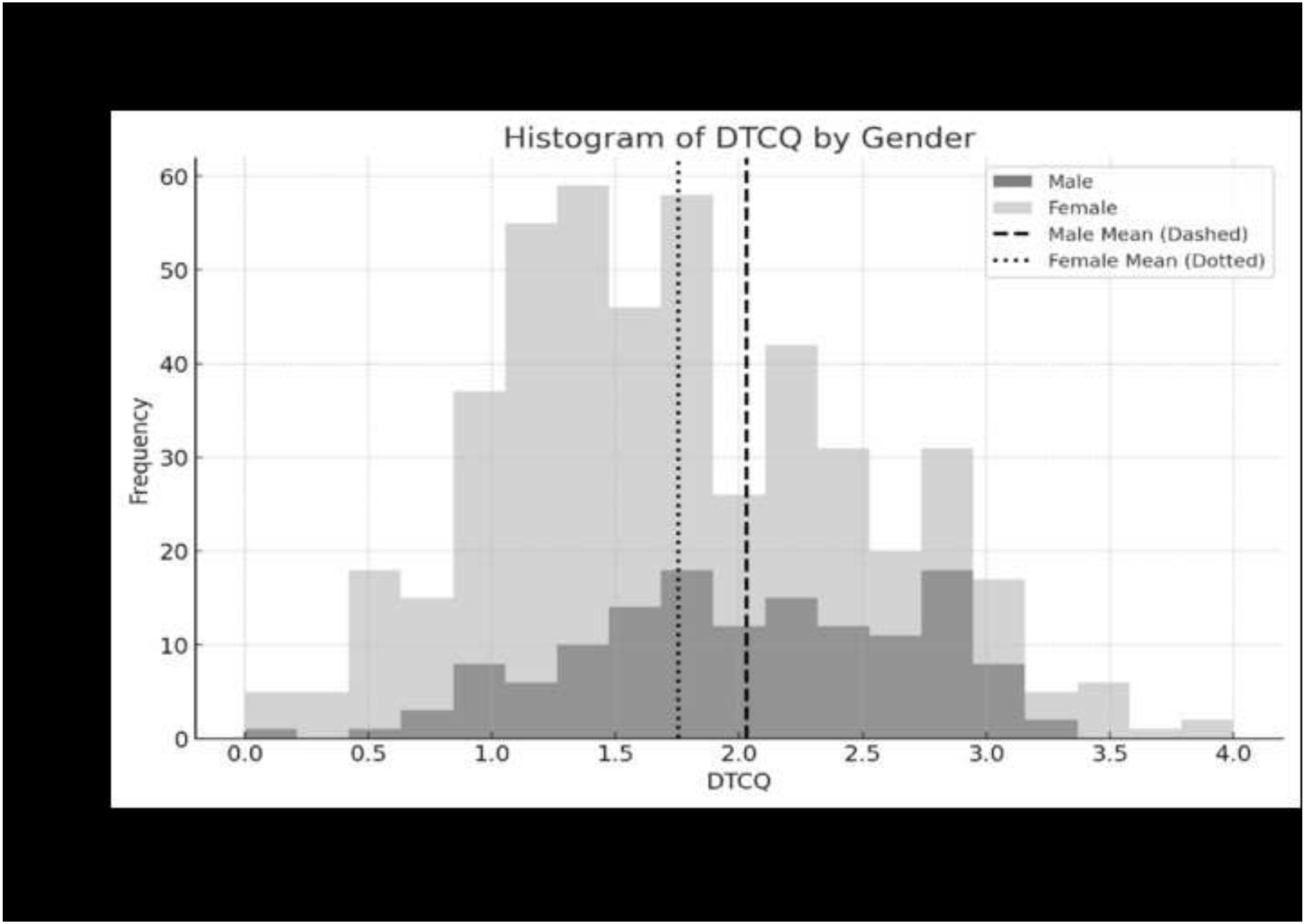

Figure
Click here to access/download;Figure;S2_Fig.tif

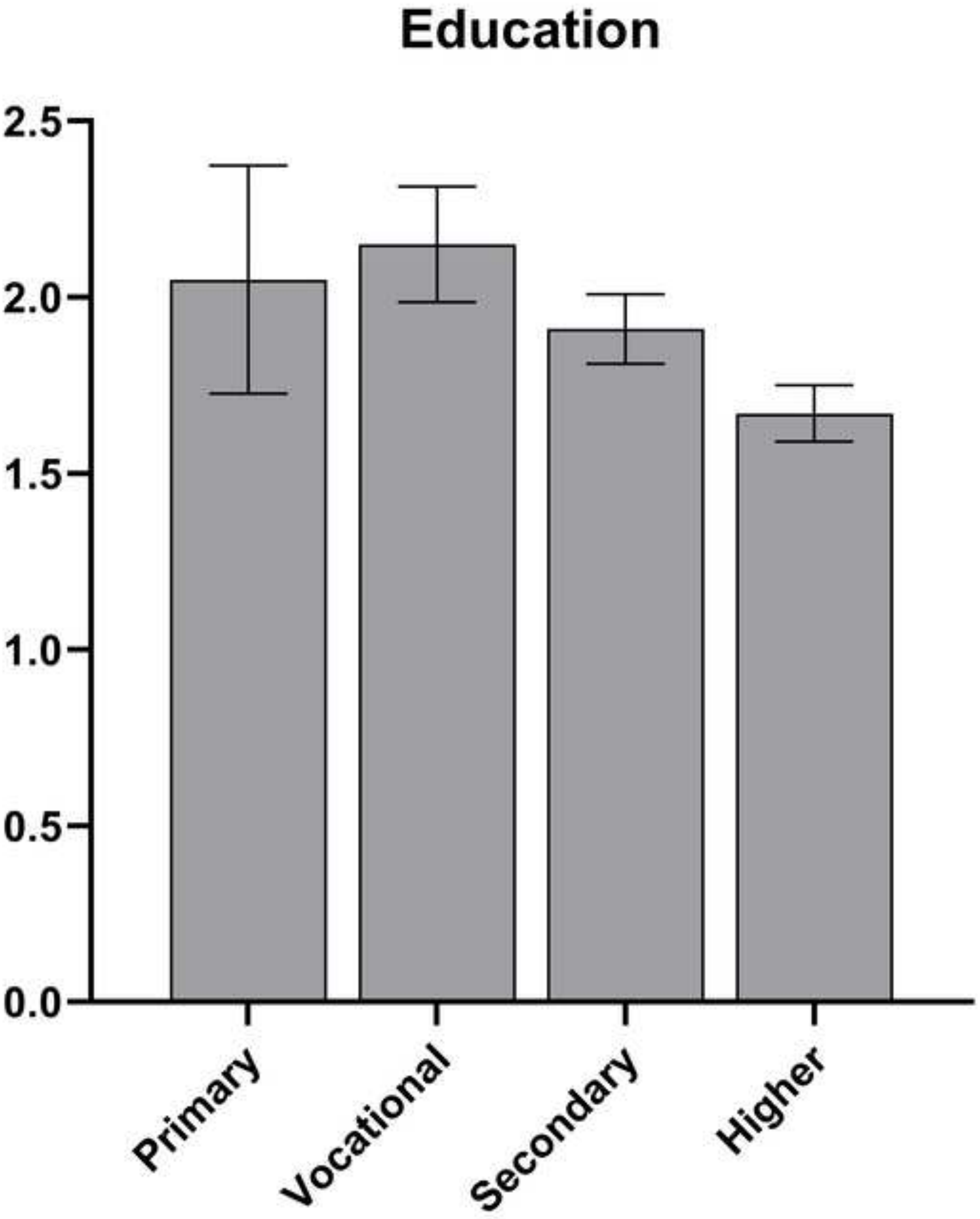

Figure

Click here to access/download;Figure;S3_Fig.tif

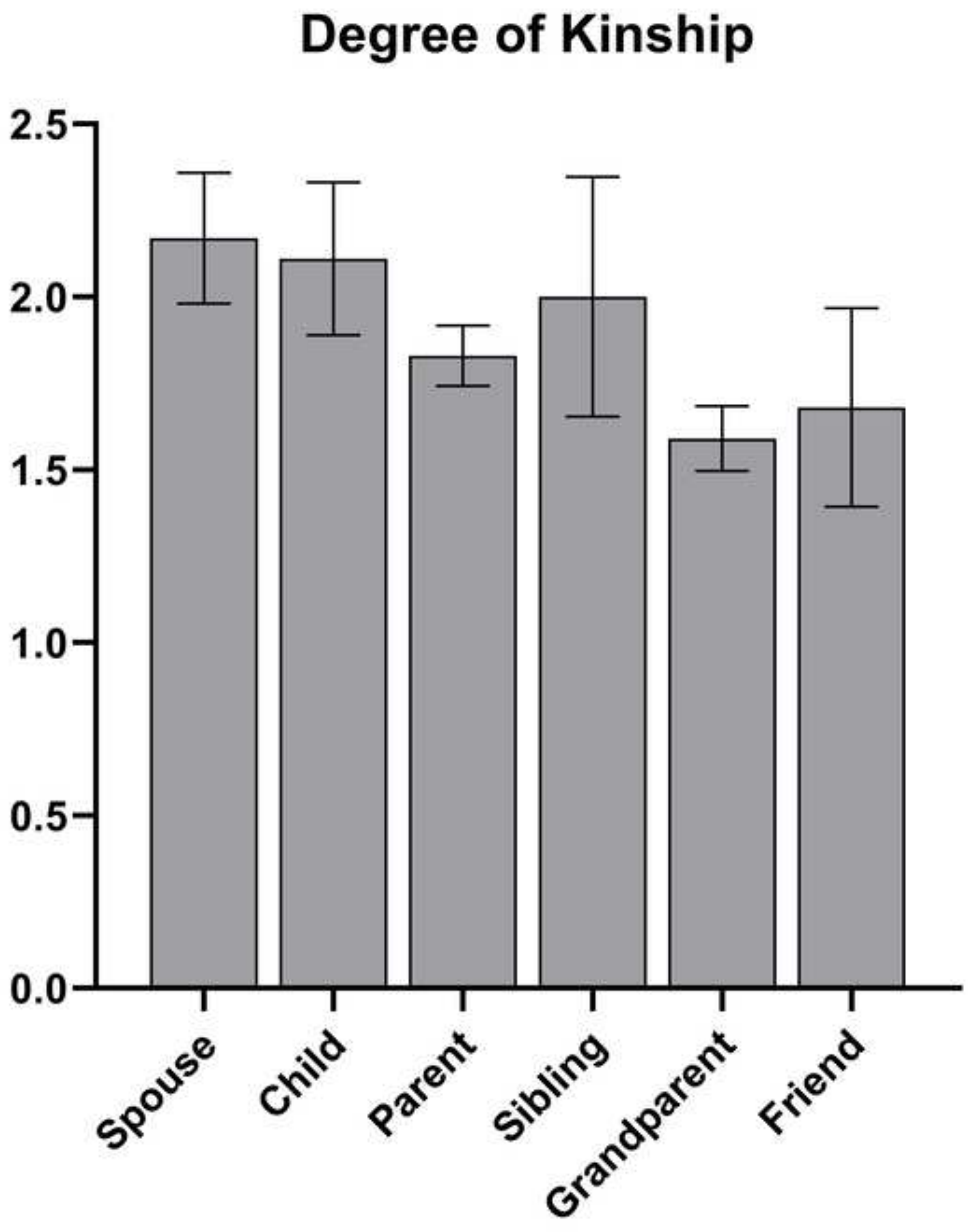

Figure

Click here to access/download;Figure;S4_Fig.tif

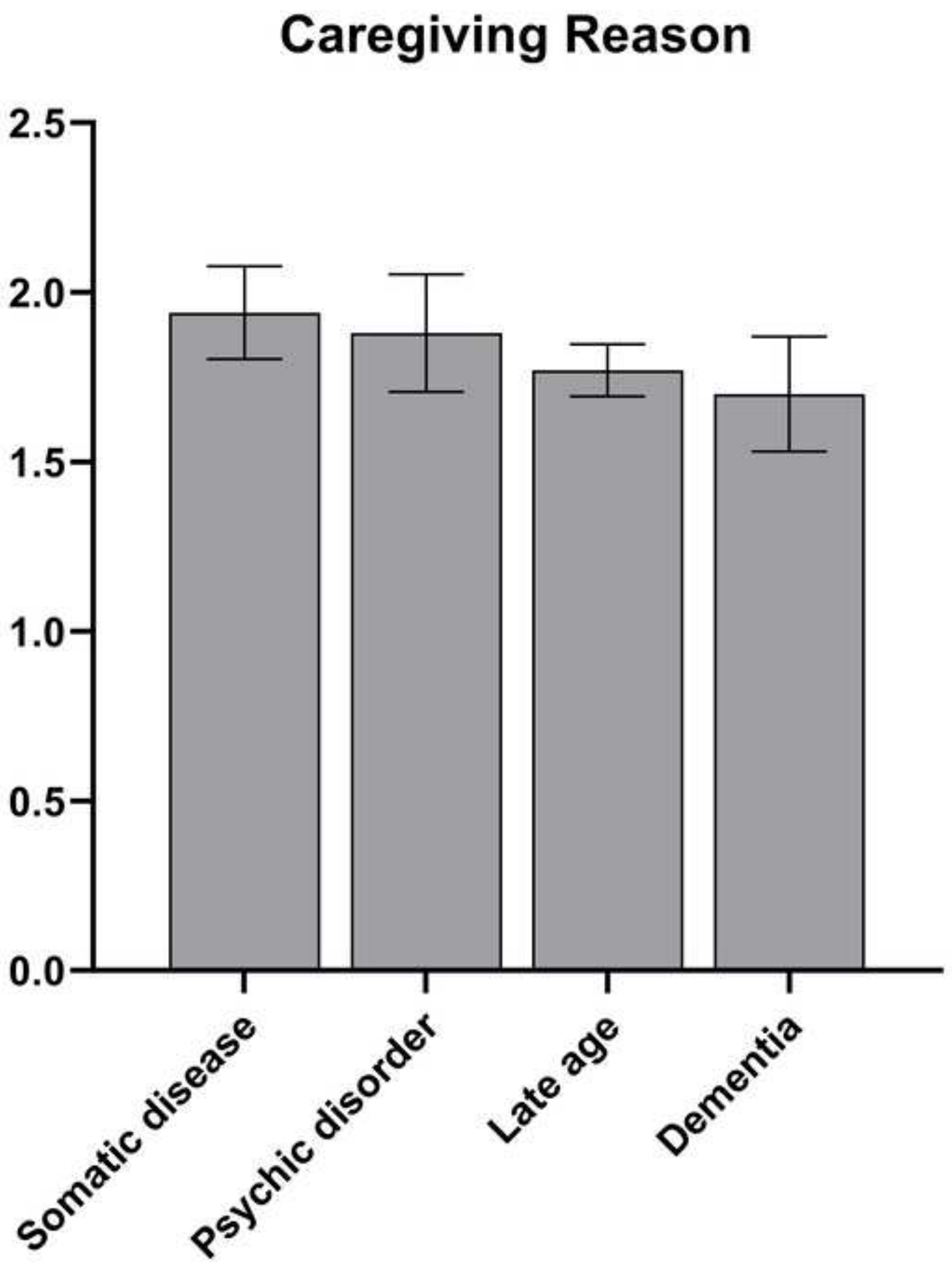

Click here to access/download
Supporting Information
S1_File.pdf

Click here to access/download
Supporting Information
DTCQ-Polish Version.pdf

Click here to access/download
Supporting Information
DTCQ English version.pdf
