# Supplementary material for: Psychometric properties of the polish version of the Dysfunctional Thoughts about Caregiving Questionnaire (DTCQ)
Source: PLoS One. 2025 May 9;20(5):e0320850. doi: 10.1371/journal.pone.0320850 (PMC12063841; doi:10.1371/journal.pone.0320850)
Supplement: Attachment — Submitted filename: Rebuttal letter (1).docx [file pone.0320850.s009.docx]

Dear Editors,

We would like to thank both reviewers for their time and valuable comments on our article. Below are the reviewers' comments and suggestions, as well as our responses.

Reviewer #1

**Abstract**

**1. In line 30, I recommend replacing the term translation with adaptation, as this more accurately reflects the comprehensive scope of work undertaken, which extends beyond a straightforward translation.**

Authors' response: Corrections have been made as suggested by the reviewer (line 31)

**Introduction**

**2. In the Introduction (lines 83–85), the authors mention various dysfunctional thought-related questionnaires used in caregiving research, identifying the Dysfunctional Thoughts About Caregiving Questionnaire (DTCQ) as one of these instruments. It would strengthen the manuscript to provide a clear justification for selecting this particular instrument for adaptation into Polish.**

Authors' response: Corrections have been made as suggested by the reviewer. We explained the choice of questionnaire in relation to the theoretical model on which it is based. The cognitive-behavioral model has been repeatedly recognized as valuable in explaining mental health disorders, and therapy based is considered to be effective (lines 94-97).

**3. In lines 101–102, the authors state that the second objective of the study was to examine the relationships between the characteristics of family caregivers, contextual factors, and caregivers' quality of life. It would be helpful to clarify whether this objective is integrated into the analysis of the psychometric properties of the instrument and the search for evidence of validity, or if it represents an independent aim of the study.**

Authors' response: Corrections have been made as suggested by the reviewer (lines 106-108).

**4. In line 125, I suggest again replacing the term translation with adaptation for the reasons stated above.**

Authors' response: Corrections have been made as suggested by the reviewer (line 131).

**5. In lines 132–135, the authors propose a hypothesis regarding the relationship between DTCQ scores, sense of identity in the caregiver role, and several contextual factors that influence caregiving. To enhance clarity, I recommend specifying the expected direction of these relationships.**

Authors' response: Corrections have been made as suggested by the reviewer. The text includes a section in which we describe our hypotheses and justify them. Regarding the relationship between dysfunctional thoughts and a sense of identity, we predicted that a strong identification with the role of caregiver, when "*being a caregiver*" is the centerpiece of identity around which only the rest of life's activities are organized, could be associated with a sense of sole responsibility and striving for perfectionism in caregiving (lines 138 -160) .

**Materials and Methods**

**Participants**

**6. It would be beneficial for the manuscript to specify the sampling procedure employed in the study.**

Authors' response: Corrections have been made as suggested by the reviewer. Thank you for your comment - we specified the sampling procedure and we have put its description in the section “Participants and Procedure” (Lines 175-189).

**7. In describing the participants' characteristics, some information is presented redundantly in both the text and Table 1 (e.g., gender and reasons for caregiving). I suggest removing the duplicate information from one of these locations. Additionally, within Table 1 itself, some rows contain repeated information, and the sample size is mentioned two times in the header. A thorough review of the table is recommended to address these issues.**

Authors' response: Corrections have been made as suggested by the reviewer.. We removed the duplicates from the table (sex) and changed the text section. We also removed rows containing repeated information (Lines 190-194; 203 -204)

**Data Analysis**

**8. On lines 252–254, the authors should outline the procedures used to analyze the associations between DTCQ scores and sociodemographic variables, including the caregiver's age, education level, and relationship/role with the person needing care. I recommend also specifying the effect size indices associated with the relationships across all analyses performed.**

Authors' response: Corrections have been made as suggested by the reviewer. We changed the last sentence to make it more extended and describe the tests in more detail (lines 276-281).

**Results and Discussion**

**9. In lines 277–279, the authors report that the internal consistency was calculated using Cronbach's Alpha, while in the Data Analysis section (lines 247–249), they state that McDonald's omega was used. This discrepancy should be resolved for consistency.**

Authors' response: Thank you for this comment. There was indeed an error in the text- reliability was tested with alpha cronbach.

**10. Effect sizes do not appear to have been adequately considered in the correlation analyses. For example, the correlation between the DTCQ general score and SCL-27 Depression, although statistically significant, is small. Similarly, the relationship with U-MICS In-Depth Exploration demonstrates a small effect size. It would be helpful to use and reference a guideline for interpreting the magnitude of such correlations.**

Authors' response: For both correlation and difference tests, we have added a guideline for interpreting the magnitude of such correlations (Cohen, 1988). We also included the results of Small effect sizes in the discussion (Lines 317 - 319; 401-405)

Reviewer #2

1. **Indent the first line of the paragraph**

Authors' response: Corrections have been made as suggested by the reviewer.

2. **Provide some examples**

Authors' response: Corrections have been made as suggested by the reviewer. In line with research results, we have given examples of the most common mental health disorders - anxiety and depression (lines 49-50).

3. **Comma (I think)**

Authors' response: Corrections have been made as suggested by the reviewer.

4. **I strongly recommend to verify mediation effects of dysfunctional beliefs on the relationships between IV variables and depressive symptoms.**

Authors' response: We would like to thank the reviewer for the very interesting suggestion. The primary aim of this study was to examine the psychometric properties of the Polish version of the DTCQ through evaluating its factor structure, reliability, and construct validity. We focused on establishing the base validity of this measure in the Polish cultural context, including testing whether the original factor structure holds and examining basic relationships with theoretically relevant variables for external validation purposes. While we agree that mediation analyses could provide valuable insights into the mechanisms linking dysfunctional thoughts with caregiver outcomes, such analyses would be premature before establishing the basic psychometric properties of the Polish DTCQ.

Our findings regarding factor structure and relationships with external variables already revealed some important cultural differences (e.g., the one-factor vs. two-factor structure) that should be well understood before moving to more complex analyses. However, now that we have validated the Polish version of DTCQ, future research could indeed benefit from examining mediational pathways - particularly in understanding how dysfunctional thoughts might mediate the relationship between caregiver characteristics and outcomes, or how identity formation might mediate the relationship between dysfunctional thoughts and caregiver adaptation. We have noted this as an important direction for future research, especially in the context of our findings regarding identity commitment and dysfunctional thoughts**.**

5. **Indent the first line of the paragraph**

Authors' response: Corrections have been made as suggested by the reviewer.

6. It suggests continuous variable. Rather: "Area of residence was classified into 5 categories"

**Authors' response:** Corrections have been made as suggested by the reviewer.

7. **There were normality tests carried out?**

Authors' response: Thank you very much for this comment. As it states in the text, the normality tests were performed:  **“**In the first step, we conducted a normality analysis of all the studied continuous variables” (Lines 264 - 266)

8. **As I mentioned earlier - mediation analysis would be valuable.**

Authors' response: **T**hank you again for this comment. We addressed this suggestion in point 4.

9. **According to APA7: "Use italics for letters used as statistical symbols or algebraic variables (e.g., contained t-test p values; R2 = .12)"**

Authors' response: Corrections have been made as suggested by the reviewer (Lines 329-345).

We would like to once again express our gratitude for all the comments. We believe the article is suitable for publication in PlosOne.
